# Generative design of singlet fission materials leveraging a fragment-oriented database

**DOI:** 10.1039/d5sc03184b

**Published:** 2025-08-25

**Authors:** Thanapat Worakul, Rubén Laplaza, J. Terence Blaskovits, Clémence Corminboeuf

**Affiliations:** a Laboratory for Computational Molecular Design, Institute of Chemical Sciences and Engineering, Ecole Polytechnique Fédérale de Lausanne (EPFL) 1015 Lausanne Switzerland clemence.corminboeuf@epfl.ch; b National Center for Competence in Research-Catalysis (NCCR-Catalysis), École Polytechnique Fédérale de Lausanne (EPFL) 1015 Lausanne Switzerland; c Max-Planck Institute for Polymer Research Ackermannweg 10 55128 Mainz Germany

## Abstract

Recently, we leveraged the FORMED repository made up of 116 687 synthesizeable molecules to deploy fragment-based high-throughput virtual screening (HTVS) and genetic algorithm (GA) searches of singlet fission (SF) molecular candidates. With these approaches, both prototypical (*e.g.*, acenes, boron-dipyrromethane (BODIPY)) and unreported (*e.g.*, heteroatom-rich mesoionic) classes of chromophore candidates fulfilling specific SF energetic requirements were identified. Yet, the reliance on predefined fragments limits chemical space exploration and, thus, the discovery of truly unforeseen molecular cores. Here, we exploit FORMED to train a generative learning framework driven by reinforcement learning and property predictions. The generative model rediscovers a diverse range of previously reported SF chromophore classes, including polyenes, benzofurans, fulvenoids and quinoidal systems, but also suggests an unexpected scaffold absent from the training data, neocoumarin (2-benzopyran-3-one), characterized by two endocyclic double bonds in an *ortho* arrangement and capped by a lactone group. An in-depth investigation reveals a diradicaloid behavior over the conjugated core comparable to 2-benzofuran, a widely known SF compound. This work highlights the potential of using both generative and property prediction models to discover candidates beyond derivatives of known chemistry for tailored material applications.

## Abstract 2

Combining the FORMED database with a generative model and the prediction of excited state propertoes, we generate molecular candidates for singlet fission (SF). Amidst known candidates, we find a promising neocoumarin (2-benzopyran-3-one) scaffold.

## Graphical abstract

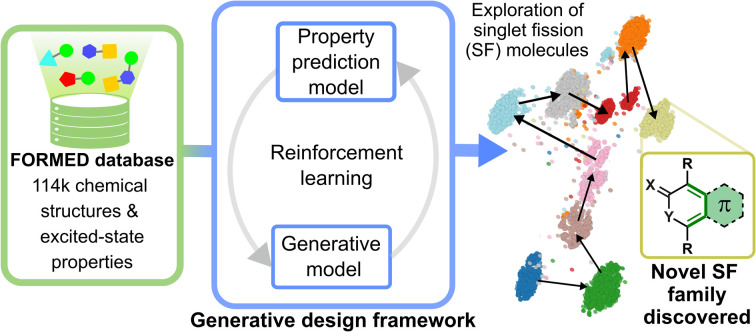

## 1 Introduction

The singlet fission (SF) process^1–4^ refers to the spin-allowed conversion of a singlet excited-state (S_1_) into two lower-lying triplet states (T_1_). This phenomenon has the potential to improve the power conversion efficiency of silicon single-junction solar cells by exceeding the Shockley–Queisser thermodynamic efficiency limit of 33.7% (ref. 5 and 6) in silicon single-junction solar cells. However, materials suitable for SF must satisfy several stringent energy-based criteria: (1) the energy of S_1_ must be at least twice or greater than that of T_1_ for the process to be thermodynamically feasible,^1^ (2) the energy of T_1_ must be higher than the conduction band of silicon (∼1.12 eV) to ensure triplet energy transfer to a semiconductor^7,8^ and (3) the S_1_ energy must align with the energy of the incoming photon, typically in the visible or near-visible range (1.5–3.5 eV).^9^ These preliminary requirements make the identification and design of suitable SF materials a challenging task.

**Fig. 1:**
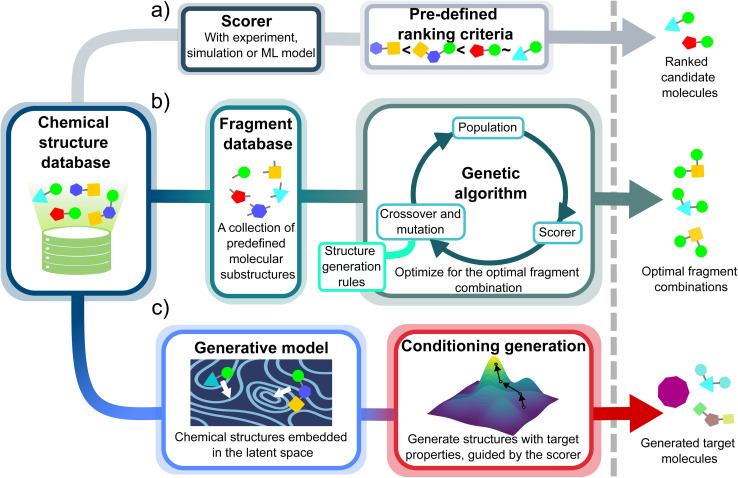
Overview of molecular design strategies: (a) high-throughput virtual screening (HTVS), which evaluates the properties of compounds in a virtual library, ranks them, and selects candidate compounds with the top properties. (b) Genetic algorithm, where the property space is explored using predefined structure generation rules in the crossover and mutation steps. (c) Data-driven generative models, which learn structure generation rules by encoding chemical structures into a latent space. Structures are generated by sampling the learned latent space. With reinforcement learning, the generative process is biased toward molecules with tailored properties.

Extensive experimental and computational work has focused on designing SF materials, primarily through the screening and modification of known compounds, leading to tailored design rules.^10–21^ In this spirit, some of us previously built the FORMED dataset by mining the Cambridge Structural Database (Fig. 1a) and characterizing 116 687 experimentally accessible organic molecules with time-dependent density functional theory (TD-DFT).^22^ FORMED enabled the construction of over a million donor–acceptor copolymers by cross-coupling the C(sp^2^) sites of selected fragments *in silico*, which were subsequently screened using statistical models to identify systems with suited SF thermodynamics. Although this approach successfully identified several potential donor–acceptor systems, it relied on previously defined heuristic rules^23^ to limit the combinatorial space.

To navigate the chemical space more efficiently, this high-throughput effort was followed by the development of an uncertainty-controlled genetic algorithm (GA),^24^ based on *NavicatGA*,^25,26^ (Fig. 1b). Upon GA optimization, molecules were assembled from a FORMED-derived pool of fragments, called reFORMED, and ensemble machine learning predictive models trained on FORMED data served to score candidates. This approach led to the rediscovery of known SF compounds and to the identification of acceptors, such as heteroatom-rich mesoionic compounds, not previously investigated for SF. However, genetic optimization requires a predefined fragment database and fixed rules for their recombination, which inherently limits the potential of genetic approaches to identify structural motifs completely outside the box.^27–29^

This limitation is potentially overcome by deep learning-based generative models,^30–37^ which implicitly learn the rules for generating chemical structures. These models, trained on molecules, uncover the underlying structural patterns and relationships among them, encoding this information into a continuous latent space, a compressed representation of molecules. By sampling from this latent space, generative models create molecules that reside within the learned chemical domain. Free from the constraints of manually predefined recombination rules and fragment libraries, generative models enable a broader, unbiased chemical space exploration, with the potential of discovering compounds that traditional approaches would have missed.

Once the generative models have learned to create molecules, multiple conditioning strategies are devised to steer the generative process toward desirable properties, thereby enabling the inverse design process.^38–44^ There are several approaches to direct the generation to the target molecules, such as gradient optimization in the latent space,^45^ gradient-based guidance diffusion,^38^ and classifier-free guidance diffusion.^46,47^ Among these, reinforcement learning (RL)^39–44^ is the optimization method that iteratively refines the model to meet target objectives. In each RL iteration, the model generates a batch of candidate molecules, which are then evaluated using a scoring function that quantifies how closely the generated molecules align with the desired properties. Based on the evaluation, the model parameters are updated through a feedback loop that improves its ability to generate higher-scoring molecules in subsequent iterations. Over multiple iterations, the generative model's outputs are refined toward compounds that satisfy the expected properties. Li and Tabor^48^ demonstrated the potential of this approach by integrating a generative model with RL to identify SF candidates. Because their methodology relied on semi-empirical computations to evaluate excited-state energies during each RL iteration and because their model was trained on a ChEMBL database^49^ containing only small drug-like molecules, the exploration of the chemical space remained limited, leading to the identification of thiophene and acene derivatives primarily. Replacing these expensive computations with machine learning models as scoring functions and leveraging a chemically diverse database to train the models offers an efficient alternative, enabling scalable and broader chemical space exploration.

Here, we develop a data-driven design platform in which both structure generation and structure–property estimation are accomplished *via* machine learning. We leverage the FORMED database to train both the generative and property prediction models (Fig. 1c). By combining a generative model capable of creating diverse molecules with a robust predictive model that estimates excited-state properties with minimal expense, our approach offers an efficient means for discovering unexpected molecular scaffolds fulfilling the energetic criteria of SF materials. Our approach specifically discovers a hitherto unknown cyclic ester core, neocoumarin not present in FORMED, that has been previously synthesized. This class of molecules follows the well-known diradical design principle elaborated by Michl and coworkers,^17^ while overcoming some of the limitations of the 2-benzofuran core. Importantly, the approach also rediscovered most other known or predicted classes of SF molecules, including acenes, polyenes, and benzofurans.

## 2 Methodology

**Fig. 2:**
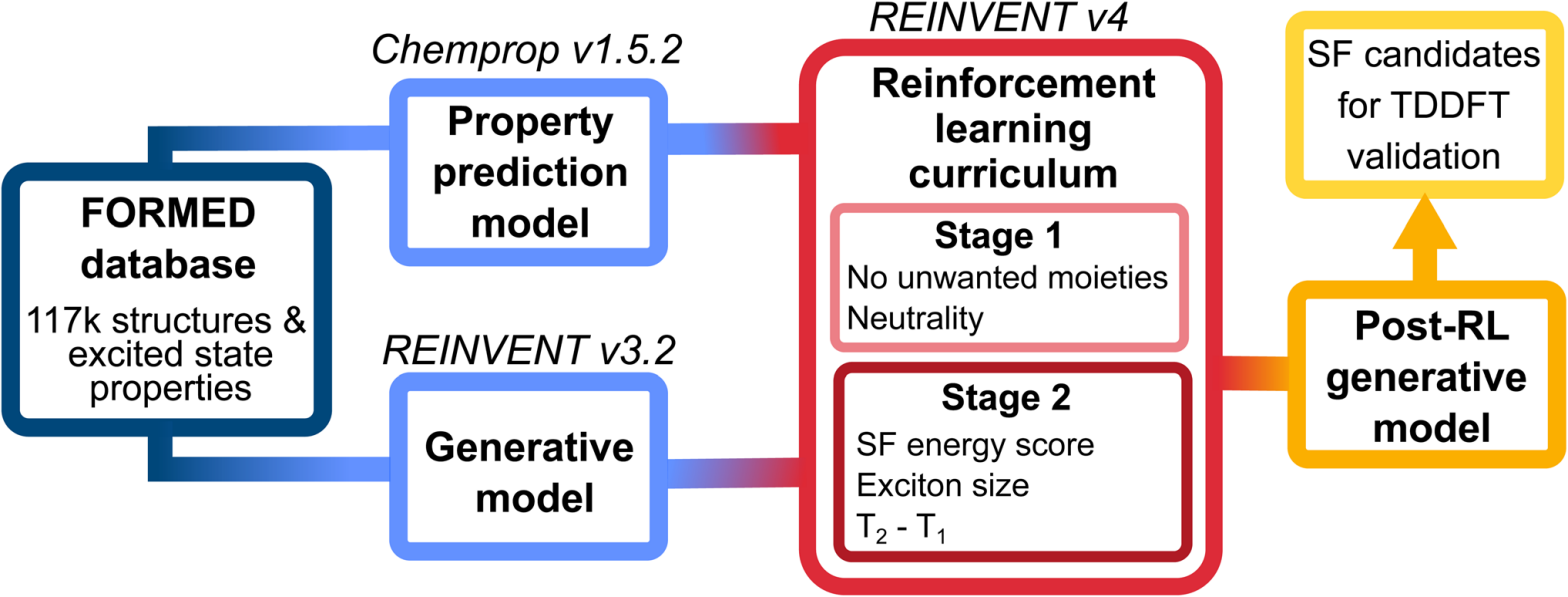
Workflow of the generative design pipeline, which incorporates three deep learning platforms: REINVENT v3.2 for training the generative model, Chemprop v1.5.2 for training the property prediction model, and REINVENT v4, which conditions the molecular generation process using a reinforcement learning curriculum approach, yielding the post-RL generative model that selectively creates molecules that meet the SF requirements. The reinforcement learning curriculum consists of two stages: the first stage focuses on structural constraints, while the second stage optimizes excited-state properties for singlet fission.

The proposed generative design workflow involves both a generative and a property prediction model (blue boxes, Fig. 2). Both models utilize SMILES strings as the molecular representation. The pre-RL generative model is first trained to generate chemically valid molecules without specific property constraints, while the property prediction model evaluates the excited-state properties of these molecules. This pre-RL generative model is then coupled with the prediction property model through Reinforcement Learning, a training framework that iteratively updates the generative model's parameters to increase the likelihood of generating desirable molecules. We implement RL in two stages, following a curriculum of increasing complexity (red boxes, Fig. 2). The resulting optimized post-RL generative model (yellow box) is able to selectively generate molecules with properties suitable for SF. In what follows, we briefly describe all three components, while additional details are provided in the SI.

### 2.1 Prediction of excited-state properties

**Fig. 3:**
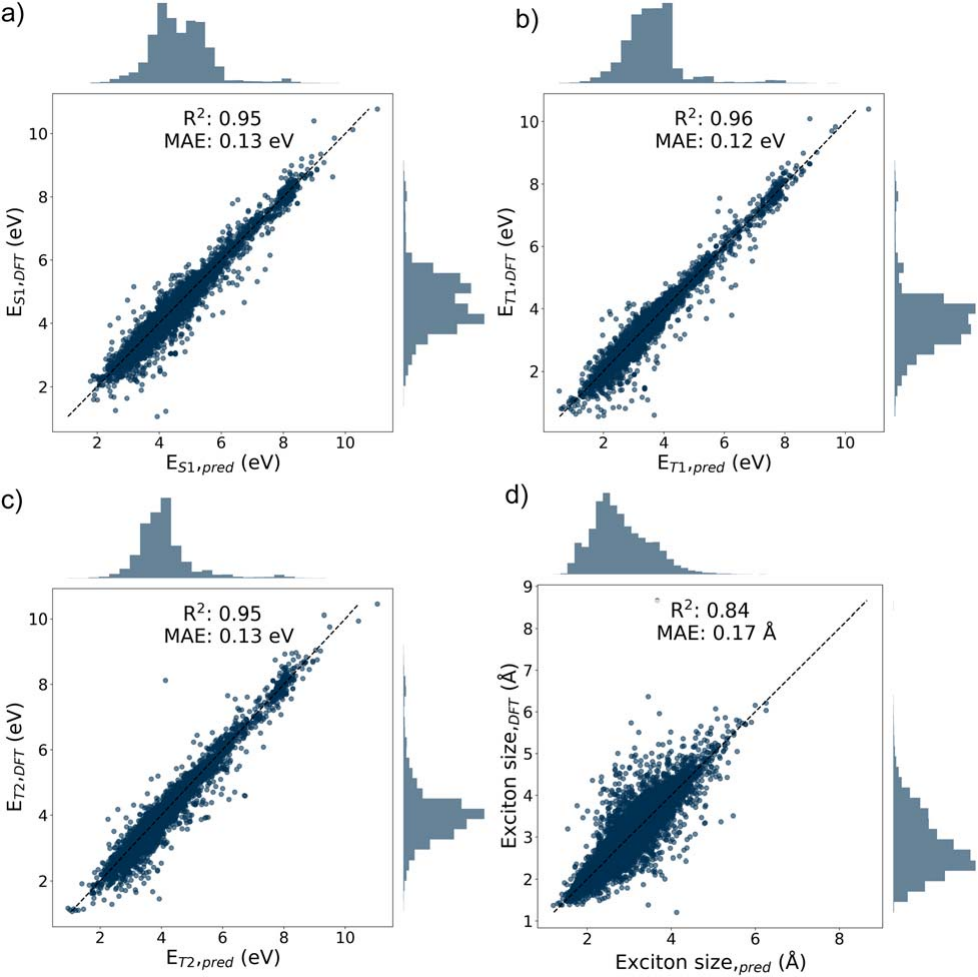
Correlation plots of excited-state properties comparing the predicted values from the Chemprop model with the true values of molecules in the test set, obtained from a random split of the FORMED database: (a) *E*_S_1_,ve_, (b) *E*_T_1_,ve_, (c) *E*_T_2_,ve_, and (d) S1 exciton size 
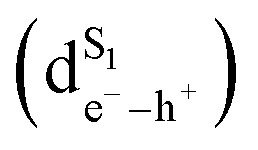
.

To predict the excited-state properties of interest, a GNN-based multi-target property prediction model was trained on the FORMED database, which consists of 116 687 molecules, along with their excited-state properties, using Chemprop v1.5.2.^50^ This model predicts four key electronic excited-state properties—singlet and triplet vertical excitation energies (*E*_S_1_,ve_, *E*_T_1_,ve_, and *E*_T_2_,ve_) as well as exciton size 
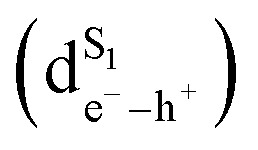
—from SMILES as a molecular representation. The Chemprop model architecture consists of a 3-layer GNN with a hidden size of 300 and a dropout probability of 0.2. The dataset was randomly split into training, validation, and test sets with an 80/10/10 ratio. On the test set, the Chemprop model achieved mean absolute error (MAE) losses of 0.13 eV for *E*_S_1_,ve_, 0.12 eV for, 0.13 eV for *E*_T_2_,ve_, and 0.17 Å for the exciton size (Fig. 3).

In line with previous work,^24^ we also evaluated the predictive performance of the Chemprop model on an external test set derived from the reFORMED database. The trained model demonstrated acceptable predictive accuracy across all excited-state energies, with MAE values of 0.22 eV for *E*_S_1_,ve_, 0.20 eV for *E*_T_1_,ve_, and 0.31 Å for the exciton size (Fig. S1). As such, we concluded that the multi-target Chemprop model is able to predict the excited-state properties of unseen molecules accurately enough for the RL process (*vide infra*).

### 2.2 Molecular generative models

Numerous generative deep learning models have been developed for creating molecules, including variational autoencoders,^51–53^ generative adversarial networks,^53,54^ flow-matching models,^55,56^ and diffusion models.^57–63^ Among these, REINVENT,^64,65^ a recurrent neural network (RNN)-based framework designed to learn and generate SMILES strings, has emerged as one of the most effective tools for molecular design applications. REINVENT is especially appealing due to its user-friendly interface. Model training and fine-tuning can be easily configured *via* TOML or JSON files. In this work, we adopt REINVENT v3.2 (ref. 64) to learn canonicalized SMILES strings from the FORMED database.^22^ In this framework, a SMILES string is treated as a sequence of tokens, where each token is either a single character or a combination of characters. A token pool is created at the start of the training process, and the model is trained unconditionally to learn the joint probability **P**(*T*) of generating a SMILES sequence *T* of length 
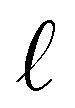
 with tokens 
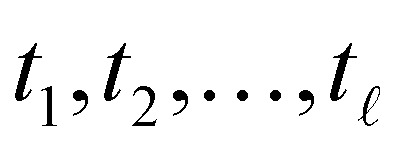
. The joint probability is expressed as:1
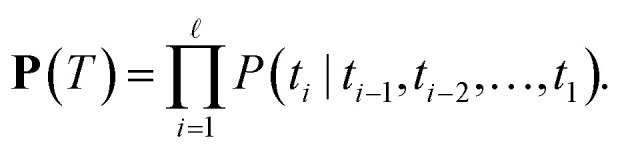


The training involves minimizing the negative log-likelihood (NLL), which quantifies how well the model predicts the sequences in the training data. The NLL is defined as:2
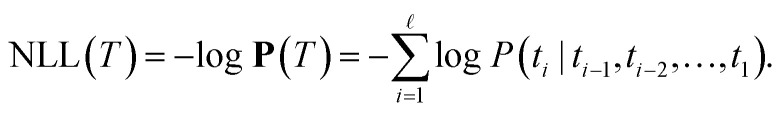


Once trained, the model generates SMILES strings by sampling tokens sequentially from the learned probability distribution **P**(*T*). Starting with an initial token (^ in our case), the model predicts the probability distribution for the next token based on the conditional probabilities it has learned. The process is repeated iteratively, with each generated token influencing the prediction of the subsequent token, until sequence generation is complete. The sequence is terminated when a predefined stop token is added ($ in our case).

### 2.3 Reinforcement learning

The trained generative model (*vide supra*), is then optimized with RL using the REINVENT v4 implementation. In the RL process, molecules sampled from the agent model (that is, the generative model that undergoes optimization) are evaluated using a scoring function that quantifies their suitability for SF based on predicted excited-state properties. The construction of the scoring function is briefly outlined in the following and is covered in detail in the SI. Following the policy gradient approach,^66,67^ these score values are used as optimization signals to adjust the generative process. Specifically, these scores are used to define the augmented likelihood (**P**_aug_) for each SMILES sequence *T* as

3 log **P**_aug_(*T*) = log **P**_prior_(*T*) + *σ***S**(*T*),

where **S**(*T*) is the reward value associated with the SMILES sequence *T*, *σ* is the hyperparameter used to scale the reward value, and **P**_prior_(*T*) is the likelihood of the SMILES sequence *T* in the prior model (the initial generative model in our case). Note that, in the case of invalid SMILES sequences, a reward value of zero is assigned, although their likelihoods are still taken into account.

To optimize the agent model, the loss function is defined as4
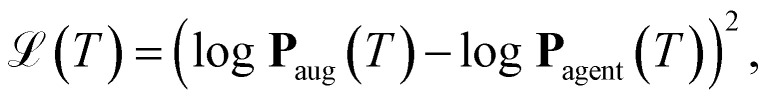
where and **P**_agent_(*T*) is the likelihood of the SMILES sequence *T* in the agent model. The presence of **P**_prior_(*T*) in **P**_aug_(*T*) serves as a regularization mechanism, constraining the agent model to remain close to the learned chemical space and ensuring the generation of chemically valid SMILES sequences. The balance between prioritizing the reward and enforcing the regularization agent model's knowledge can be controlled with the hyperparameter *σ*.

During the optimization loop, the RL tends to overexploit specific SMILES sequence patterns, corresponding to high reward value, which leads to the generation of structures with similar scaffolds within an iteration loop. To promote structural diversity among the molecules generated from the agent model, we employed the diversity filter implemented in REINVENT v4.^65^ This filter penalizes SMILES strings that are too similar to those already stored in a memory bucket, which keeps track of previously generated molecules. By discouraging the agent model from repeatedly generating structurally similar compounds, the filter ensures greater diversity. In addition, we used experience replay^68–70^ to improve the convergence of the RL process by storing high-scoring molecules generated during previous iterations and periodically reintroducing them into the training process.

The energy score function taken from previous work,^24^ where a higher score corresponds to a higher reward, was used to optimize the S_1_/T_1_ energy levels to satisfy three SF requirements:

(1) Thermodynamic constraint: *E*_S_1_,ve_ − 2*E*_T_1_,ve_ > −1 eV.

(2) Solar cell semiconductor compatibility: *E*_T_1_,ve_ > 1.5 eV.

(3) Matching with the solar emission spectrum: *E*_S_1_,ve_ < 3.8 eV.

A detailed mathematical definition of the energy score is given in the SI. In addition to optimizing the energy levels of S_1_ and T_1_, our goal was to maximize the exciton size (*i.e.*, the root-mean square electron–hole separation) to promote delocalized singlet exciton formation. A larger exciton size is indicative of charge-transfer or delocalized excited-state character, which are both beneficial for the triplet-pair formation from the singlet state. We also consider the energy gap between the vertical second and first excited triplet states, which is to be maximized to reduce the likelihood of competing T_1_ to T_2_ upconversion processes.^71^ Furthermore, we bias the generative model against the generation of charged structures by penalizing the score of charged molecules.

Given the complexity of the score function, which involves multiple objectives to be optimized simultaneously, we adopted a two-stage curriculum for the RL optimization process (Fig. 2).^72^ In the first stage, we focused on structural constraints (more details are given in the SI). To avoid overfitting, this first stage of RL was limited to 20 iterations. In the second stage, we focus on optimizing the SF-related properties, namely the energy score, exciton size, and T_1_/T_2_ gap (*E*_T_2_,ve_ − *E*_T_1_,ve_). Splitting the RL into two stages focused on different aspects significantly simplifies the learning. During each stage, the individual components of the score function were aggregated using a weighted geometric mean:5
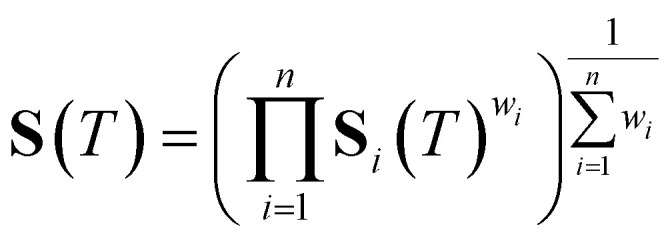
where **S**_*i*_(*T*) is the individual score component *i* for the SMILES sequence *T*, and *w*_*i*_ is the weight assigned to the *i*-th score component. Additional details regarding the implementation of the different objectives and the weighting strategy are given in Table S1.

At the end of the second stage of each trial, 1280 molecules were generated using the post-RL generative model, and their excited-state properties were predicted using the Chemprop model. Subsequently, the 10 best molecules, ranked according to their energy scores, were selected for further validation and optimized with DFT to have their properties evaluated using TD-DFT computations.

### 2.4 Computational details

The SMILES strings created with the generative models were converted into 3D geometries using the distance geometry approach implemented in RDKit,^73^ followed by refinement of atomic positions with the MMFF94 force field.^74,75^ These initial geometries were optimized at the GFN2-xTB^76,77^ level, providing an initial 3D geometry for subsequent gas phase density functional theory (DFT) geometry optimization at the ωB97X-D^78^/6-31G(d)^79–81^ level. Using the same functional and basis set, TD-DFT computations with Tamm-Dancoff approximation^82^ were carried out to determine vertical excitation energies and to perform excited-state geometry optimizations to extract adiabatic excited-state energy values. The diradicaloid character was assessed by computing open-shell singlet wavefunctions at the UHF/6-31G(d) level on the DFT optimized geometries and extracting the diradical character (*y*_0_) and the tetraradical character (*y*_1_) diagnostics as introduced by Nakano and coworkers.^24,83–85^ All electronic structure computations were performed using the Gaussian16 (ref. 86) (revision A.03) software package.

## 3 Results and discussion

### 3.1 Exploration of chemical space

**Fig. 4:**
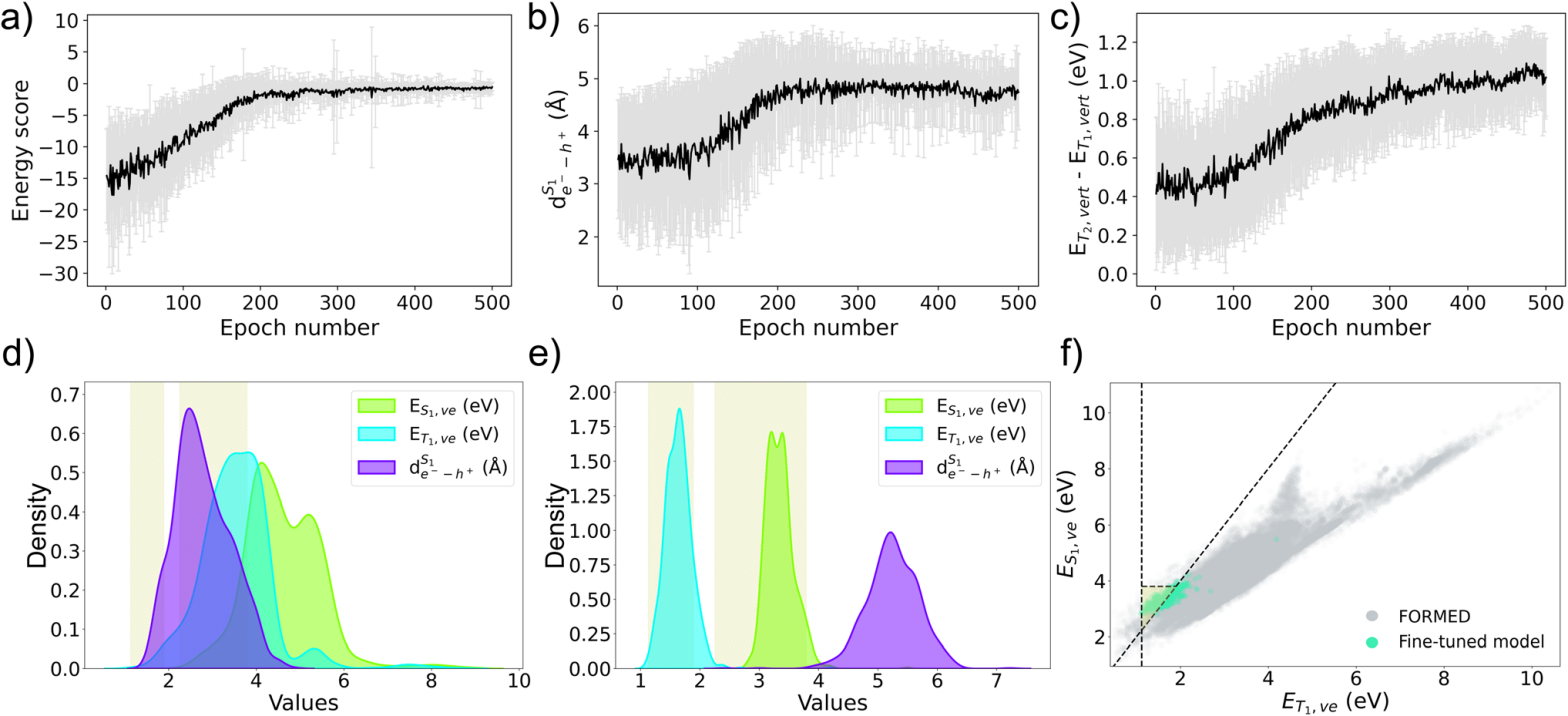
RL optimization curves during stage 2 of the curriculum for (a) energy score, (b) exciton size 
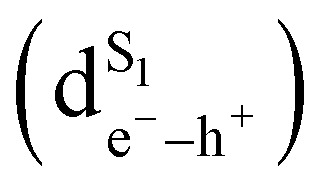
, and (c) *E*_T_2_,ve_ − *E*_T_1_,ve_. Kernel density histogram plots of *E*_T_1_,ve_, *E*_T_1_,ve_, and 
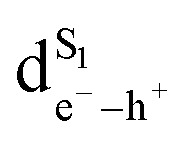
 predicted with Chemprop model for 1280 molecules generated from (d) the pre-RL generative model and (e) the post-RL generative model 1. The yellow region designates where S_1_ → 2T_1_ conversion is thermodynamically feasible and *E*_T_1_,ve_ is aligned for potential integration into solar cell applications. (f) Property map between *E*_T_1_,ve_ and *E*_T_1_,ve_ of the 1280 generated structures from the post-RL generative model 1 (blue) overlaid on top of the FORMED database (gray). Note that energy values of the generated structures in the map are predicted with the Chemprop model.

To identify potential candidates for SF, we follow the workflow shown in Fig. 2 and optimize a generative model. In the RL optimization loop, we rely on the Chemprop property prediction model for fast and inexpensive scoring. Within 600 iterations, the overall score, the energy score, and 
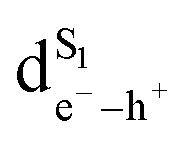
 of the generated molecules reach convergence (Fig. 4a, b and S5). The *E*_T_2_,ve_ − *E*_T_1_,ve_ is also sufficiently high (∼1.2 eV) to suppress undesired triplet–triplet upconversion (Fig. 4c). For simplicity, we narrow the discussion to the energy score and the singlet and triplet excitation energies, which are the most critical factors for SF propensity. To evaluate the impact of reinforcement learning, we compare the average excited-state properties of molecules generated before and after the initial optimization trial. Specifically, we sample 1280 molecules from both the pre-RL and post-RL generative models (model 1) and analyze their chemical structures and predicted excited-state properties.

**Fig. 5:**
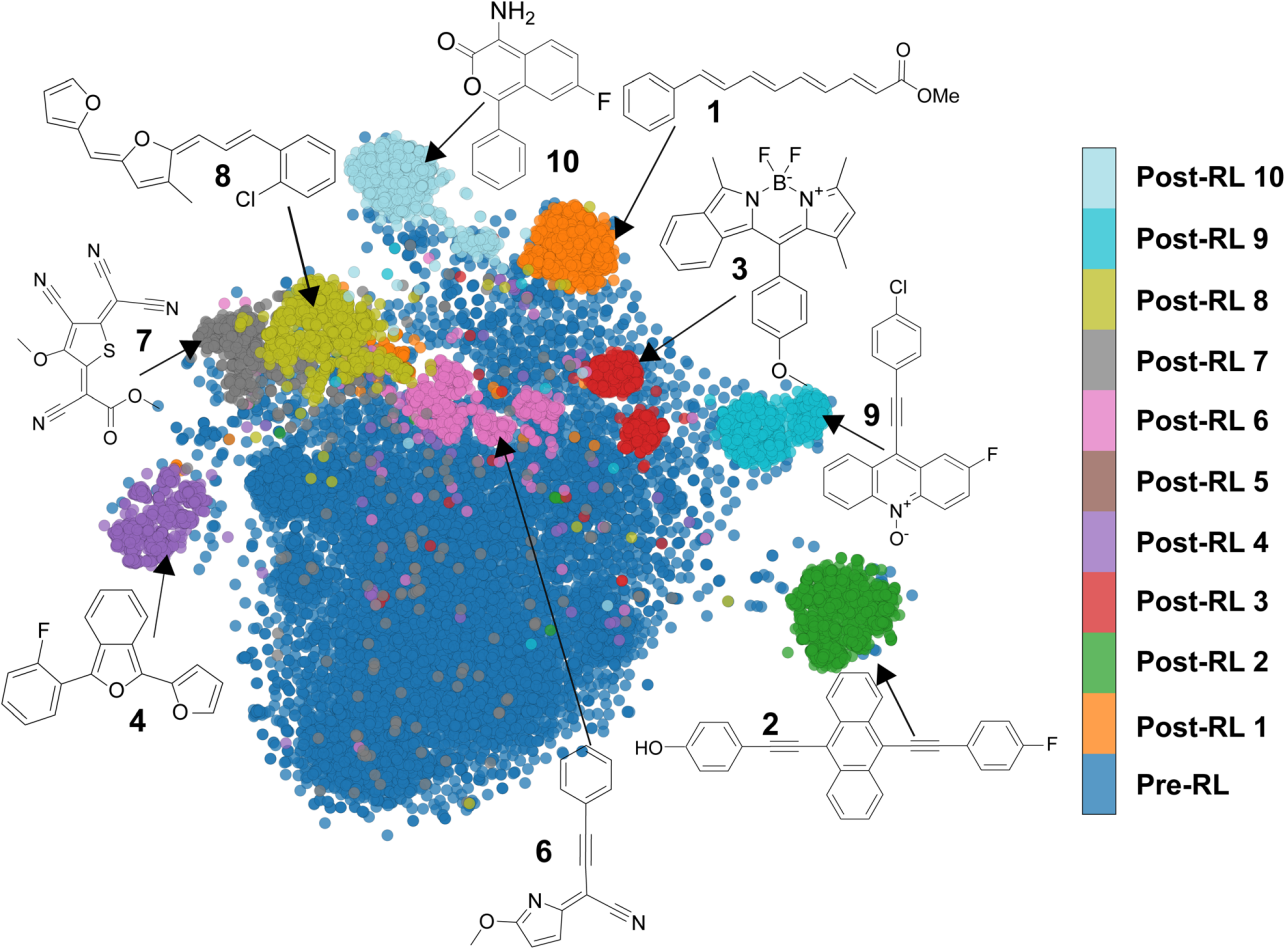
*t*-SNE plot generated from the Morgan fingerprint representation of the generated structures from the pre-RL and post-RL generative models.

The pre-RL generative model creates molecules that broadly span the chemical space of the FORMED database (Fig. S8). The resulting molecules are chemically diverse, exhibiting an average Tanimoto similarity score of 0.10 and yielding ∼367 unique scaffolds^87^ from a sample of 1280 generated molecules (Table S3). Unique scaffolds are defined as a Murcko scaffold with a Tanimoto similarity below 0.70 relative to other Murcko scaffolds in the dataset. In terms of their excited-state properties, the distributions of vertical excitation energies for the singlet (*E*_T_1_,ve_) and triplet (*E*_T_1_,ve_) states are largely overlapping (Fig. 4d), suggesting that most generated molecules do not meet the thermodynamic requirements for SF. Of the 1280 molecules generated by the pre-RL model, only two are predicted to satisfy the energetic criteria, a low hit rate at this stage. In contrast, molecules generated by the post-RL generative model 1 are confined to a narrow region of chemical space (Fig. 5) and are less diverse, with an average Tanimoto similarity score of 0.35. These molecules mainly share the same Murcko scaffolds (1–5 unique scaffolds among 1280 generated molecules, Table S3). However, the distribution of their excited-state energy levels is well separated (Fig. 4e), with the *E*_T_1_,ve_ centered at approximately half *E*_S_1_,ve_. Consequently, more than 750 structures of the 1280 molecules created by the post-RL generative model 1 are within the energetic target region for the desired SF property (Fig. 4f).

**Fig. 6:**
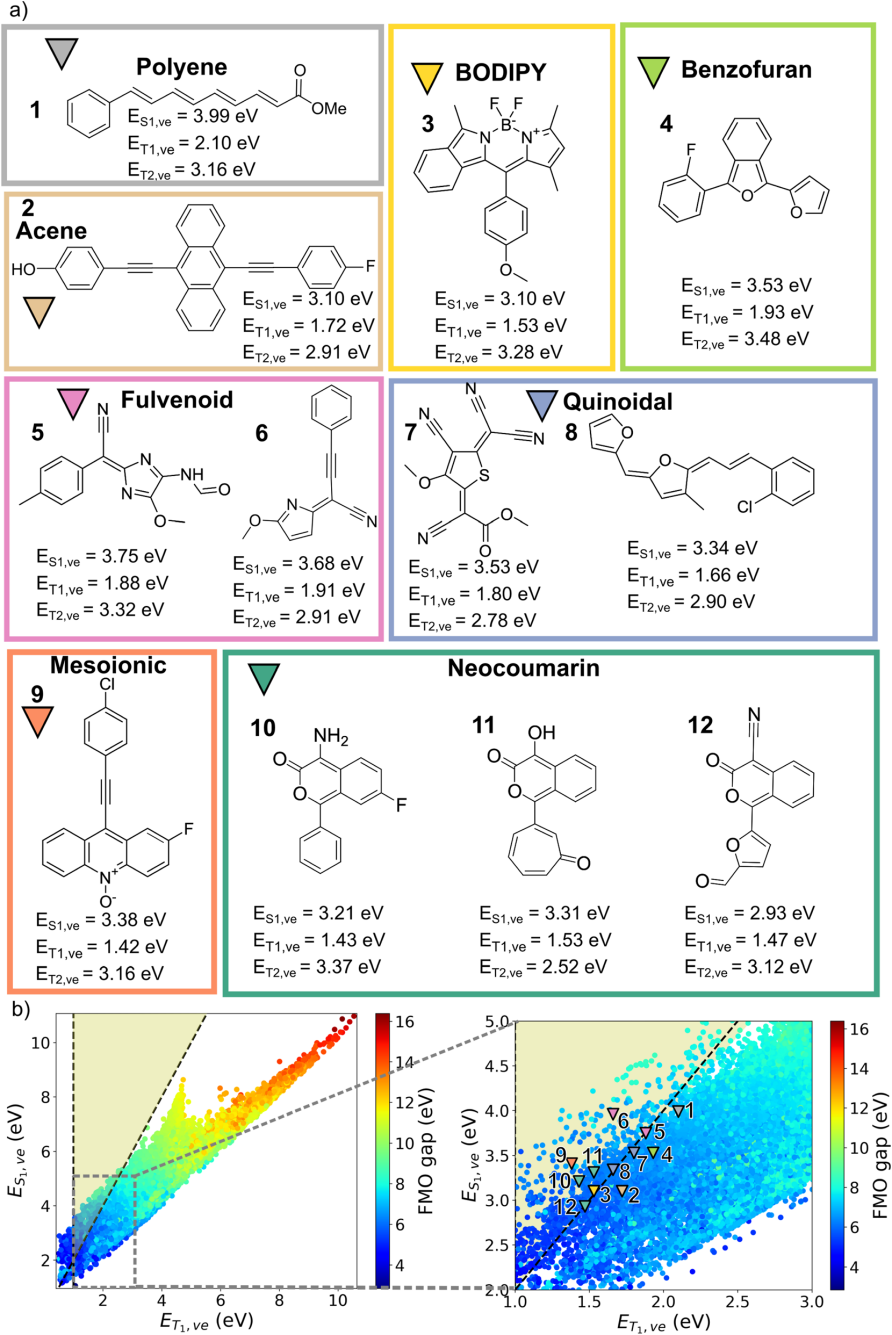
(a) Candidate molecules generated by the post-RL generative models from different optimization trial runs. *E*_T_1_,ve_ and *E*_T_1_,ve_ are computed using TD-DFT. (b) Property map between *E*_T_1_,ve_ and *E*_T_1_,ve_ of structures in the FORMED database colored by frontier molecular orbital (FMO) gap. The yellow region designates where S_1_ → 2T_1_ conversion is thermodynamically feasible. The corresponding adiabatic excitation energies and chemprop-predicted vertical excitation energies of these structures can be found in Fig. S12.

Despite improving the excited-state properties, the post-RL generative model 1 predominantly suggests polyenes (*e.g.*, 1 in Fig. 5 and 6) and similar molecules with extended π-conjugated systems that are known SF chromophores. This apparent preference for polyenes is sound considering the simplicity of their SMILES pattern, which consists of frequently occurring tokens in the FORMED database (*e.g.*, C and =). Furthermore, these molecules are abundant in FORMED (used to train both the generative and property prediction models).

To steer the generative exploration toward other chemical space regions, we introduce custom alerts,^39^ SMARTS-based filters for unwanted substructures into the scoring function at both stages of the RL curriculum. If a generated molecule contains any substructure from the predefined list, its score is set to zero, effectively penalizing the generation of such molecules. Molecules containing ring systems and extensive π-conjugated frameworks (entries 1–15 in the complete list of unwanted substructures provided in the SI) were initially penalized.

With these structural constraints, the second and third optimization trials guide the generative models toward more compact and tunable molecules featuring rigid π-conjugated cores. However, each post-RL generative model continues to create molecules within the same structural class. Specifically, the second and third post-RL models predominantly generate substituted acenes^88–93^ (*e.g.*, 2) and derivatives of boron-dipyrromethane (BODIPY)^94–97^ (*e.g.*, 3), respectively. Notably, despite these structural constraints, the diversity of molecules generated in these trials remains comparable to that of the first RL trial in terms of the number of unique scaffolds and similarity scores (Table S3), where no custom alert filter was used. Similar to polyenes, these molecule classes have been investigated for SF and are prominently represented in FORMED, with BODIPY and anthracene appearing 621 and 928 times, respectively.

We thus include BODIPY and acene in the unwanted structure list (entries 16–17 in the complete list of unwanted substructures provided in the SI) for further exploration. Incorporating the full list of unwanted substructures in the structural constraints leads to a more challenging optimization, requiring a larger number of iterations to improve the excited-state properties of the generated molecules (Fig. S4 and S5). Seven additional optimization trials were carried out with the full list of unwanted structures. Each trial directs the generative model toward a distinct family of SF chromophores, corresponding to a unique region of chemical space, as illustrated by the dimensionality reduction plot in Fig. 5. The RL optimization curves and kernel density histogram plots of the excited-state properties of all trials are presented in Fig. S3–S5, respectively. A summary of the performance of all generative models is provided in Tables S3 and S4.

At the end of each of the 10 optimizations, we collect the top 10 candidates from 1280 molecules created by the post-RL generative model according to their predicted energy scores. These top performers are then optimized with DFT, and their vertical energies computed with TD-DFT. Among the 100 evaluated molecules, 73 meet the SF criterion, demonstrating that Chemprop reliably guides the generative model toward high-reward regions of chemical space *via* RL. From this pool of computationally validated SF molecules, we (re)discover molecular classes with energy splittings that satisfy the SF thermodynamic criteria. One to three representative molecules from each post-RL generative model are presented in Fig. 6. Their adiabatic excited-state energies, along with the Chemprop-predicted vertical excitation energies, are provided in Fig. S10 and S11, respectively. The computed *E*_S_1_,ve_ and *E*_T_1_,ve_ of our candidate molecules fall within the thermodynamically favorable region for S_1_ → 2T_1_ conversion. Furthermore, these candidates' *E*_T_1_,ve_ value is adequate for their potential integration into solar cell applications (Fig. 6) and the T_1_–T_2_ energy gaps are large enough to suppress unwanted triplet upconversion. As stated above, the three initial trials coincide with the rediscovery of polyenes (1), acenes (2), and BODIPY (3). In the following seven runs, which exclude the full list of unwanted substructures, the generative model uncovered derivatives such as substituted 2-benzofurans (4), which were initially screened and identified by Michl and coworkers^17^ as potential SF chromophores. Similarly to their findings, our candidates feature substitutions at the C1 and C3 positions,^98–100^ lowering the triplet energies to better align with the energy requirements for solar cell applications than the unsubstituted counterparts (Fig. S14).

We also encountered a variety of fulvenoid and quinoidal compounds (5–8), recently identified as promising SF scaffolds.^12,101^ The fulvenoid derivatives feature diverse heterocyclic rings such as furan, thiophene, imidazole, and hydantoin, linked to aromatic rings *via* an exocyclic bridge containing one or more methine moieties, often decorated with a cyano group. Similar to the substituted fulvenes previously identified,^12^ these fulvenoid structures maintained a favorable *E*_S_1_,ve_ : *E*_T_1_,ve_ ratio. While these structures merit further investigation, they will not be the focus of the remainder of this work.

In line with a previous work by some of us, the post-RL generative model 9 yielded molecules containing mesoionic *N*-oxide motifs (9).^102^ Mesoionic heterocycles have been identified as good acceptor units for charge transfer in donor–acceptor systems.^14,21,24^ Interestingly, structures embedding the *N*-oxide in an anthracene core exhibit lower *E*_T_1_,ve_ and similar *E*_S_1_,ve_ compared to the substituted acenes, which increases the splitting (*E*_T_1_,ve_ = 1.4 eV, and *E*_T_1_,ve_ = 3.4 eV).

Overall, the diversity of the (re)discovered singlet fission chromophore candidates demonstrates the capability of the optimization pipeline, powered by a tailored scoring function and the FORMED database, to identify SF molecules across different structural classes. Furthermore, the successive inclusion of unwanted substructures as a penalty term in the RL step underscores the flexibility of the workflow.

### 3.2 A coumarin isomer as a promising scaffold

Along with the above finding, a series of coumarin derivatives sharing the same 2-benzopyran-3-one core (10–12) and emerging from the generative model 10 caught our attention, as, to the best of our knowledge, such systems have not yet been explored for SF applications. We term this scaffold neocoumarin to distinguish it from the well-known coumarin (1-benzopyran-2-one) and from its primary constitutional isomer, isocoumarin (2-benzopyran-1-one). Akin to coumarin and isocoumarin, neocoumarin derivatives feature a bicyclic system consisting of a benzene ring fused to a 2-pyrone ring.

The derivatives selected by the model include amine and aryl substituents on the pyrone ring. Substituted neocoumarin structures (10 and 11) generated by our optimization trials exhibit proper energetics for SF, with *E*_T_1_,ve_ ≈ 1.4 eV and *E*_S_1_,ve_ ≈ 3.0 eV. The distribution of the excited-state character, as visualized through the hole and electron densities derived from the natural transition orbitals (Fig. S15), shows significant overlap between the singlet and triplet states, both being localized near the aromatic subsystem of the fused ring core.

**Fig. 7:**
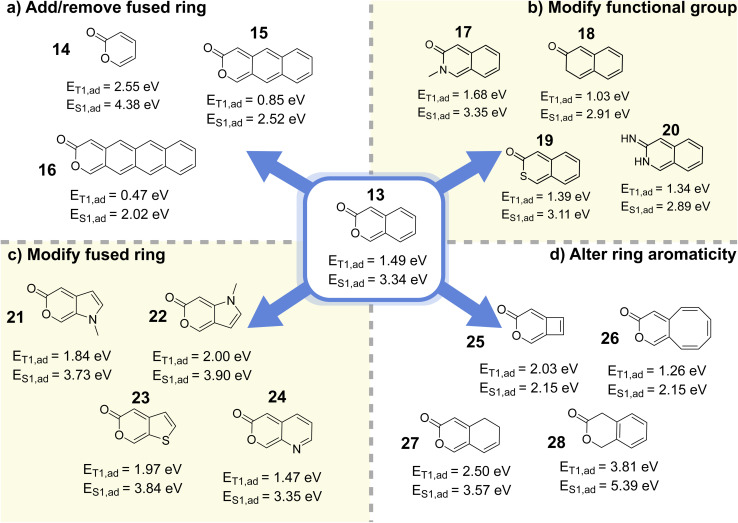
Derivative structures of neocoumarin from (a) adding or removing fused rings, (b) modifying the capping functional group, (c) switching the benzene ring to other aromatic fused rings, and (d) altering the aromaticity of the conjugated system. Structural changes that preserve excited-state energies that satisfy SF criteria are indicated by the yellow background.

**Fig. 8:**
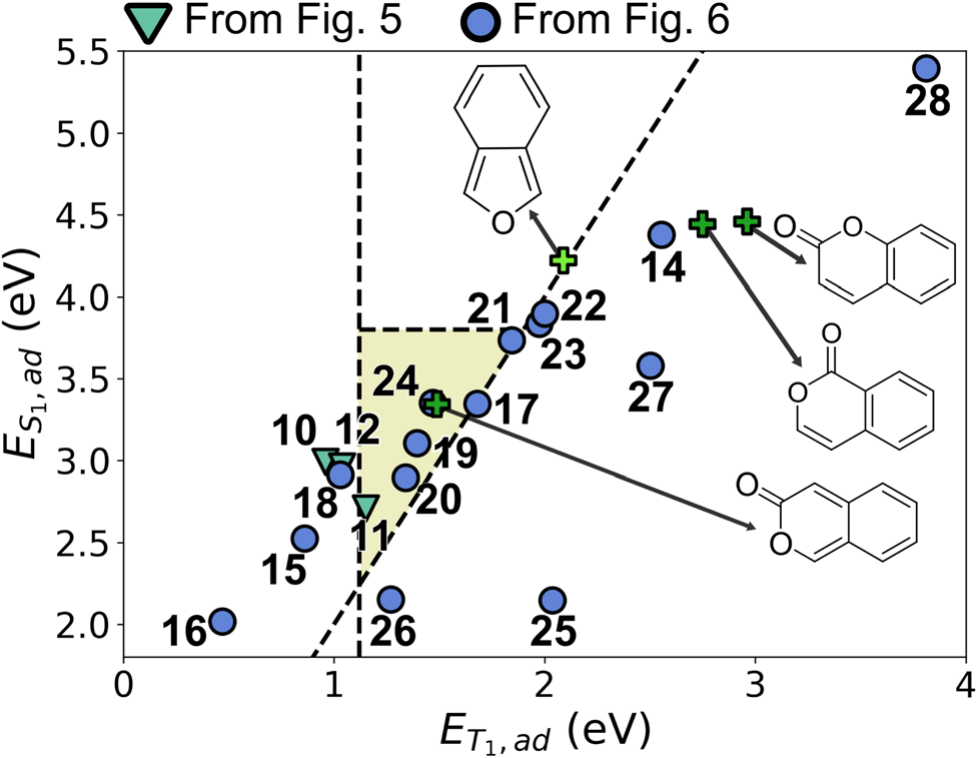
Property map between *E*_T_1_,ad_ and *E*_S_1_,ad_ of the neocoumarin-related structures. The yellow region designates where S_1_ → 2T_1_ conversion is thermodynamically feasible and *E*_T_1_,ve_ is aligned for potential integration into solar cell applications.

To identify the molecular core responsible for the SF-relevant properties and extract concrete design principles, we performed adiabatic TD-DFT computations on the bare neocoumarin structure (13, Fig. 7), which confirmed good S_1_/T_1_ splitting (*E*_T_1_,ad_ = 1.49 eV and *E*_S_1_,ad_ = 3.34 eV). We also noted that the synthesis of neocoumarin and derivatives thereof has been accomplished in a number of previous works.^75,103–105^ Interestingly, while coumarin (1-benzopyran-2-one) and isocoumarin (2-benzopyran-1-one) motifs appear 889 and 121 times, respectively, in the FORMED database, neocoumarin (2-benzopyran-3-one) is not present. Neither coumarin (*E*_T_1_,ad_ = 2.75 eV, *E*_S_1_,ad_ = 4.43 eV) nor isocoumarin (*E*_T_1_,ad_ = 2.46 eV, *E*_S_1_,ad_ = 4.43 eV) exhibit energies which are conducive to SF (green crosses in Fig. 8; see also Fig. S14). This implies that the optimization strategy succeeded at finding an out-of-sample, synthesizable chemical motif with good SF properties in spite of its absence from the training data and the inadequacy of its closest constitutional isomers.

The synthetic viability of neocoumarin opens the door to a whole class of compounds with shared structural and electronic characteristics. In order to exhaustively identify beneficial modifications and rule out modifications that disturb the SF energetics, we systematically explored the chemical space around the neocoumarin core by manually constructing diverse derivatives and computing their excited-state properties using adiabatic TD-DFT (Fig. 7). Vertical T_1_ and T_2_ excited state energy levels of these molecules are reported in Fig. S13.

We first varied the number of fused rings in the system. Removing the fused benzene ring increases *E*_S_1_,ad_ to 4.38 eV (14), while extending the number of fused rings reduces *E*_T_1_,ad_ to 0.86 eV for two fused benzene rings (15) and 0.47 eV for three fused benzene rings (16), leading to poor SF thermodynamics in both cases, thus establishing that an energetic sweet spot is achieved with two rings. The role of the lactone moiety was then explored by swapping the ester functional group in the lactone ring with an amide (17, forming a lactam), which lead to an increase of *E*_T_1_,ad_ by around 0.2 eV, and thus a poorer splitting. Replacing the lactam with a thioester instead (19) lowers *E*_T_1_,ad_ by 0.1 eV while *E*_S_1_,ad_ decreases by about 0.2 eV, which keeps the splitting ratio approximately constant. These results corroborate that the *ortho*-quinoidal double bonds are key to the photophysical properties of the system, whereas the nature of the lactone-type endcapping functional group and substituents on the pyranone unit are synthetic handles of interest for potential kinetic stability or synthesizability reasons.

The choice of ring system was also explored by manually constructing and testing different 5- and 6-membered aromatic heterocycles as a replacement for benzene to understand the limitations of this strategy. With 5-membered heterocyclic rings such as pyrrole (21, and 22), thiophene (23), the *E*_S_1_,ad_ : *E*_T_1_,ad_ ratio remains close to ideal while *E*_T_1_,ad_ increases to around 2.0 eV. However, replacing benzene with pyridine (24) has a negligible effect on SF energetics.

Since replacing benzene with other aromatic rings proved to be a viable strategy to fine-tune the neocoumarin core, we tested the role of aromaticity by using non-aromatic or anti-aromatic fused rings instead. Replacing benzene by cyclobutene or cyclooctatriene (25–26) leads to a dramatic lowering in *E*_T_1_,ad_, while breaking the delocalized π system through saturation (27–28) leads to a consistent increase in *E*_T_1_,ad_, in both cases hampering SF energetics.

**Fig. 9:**
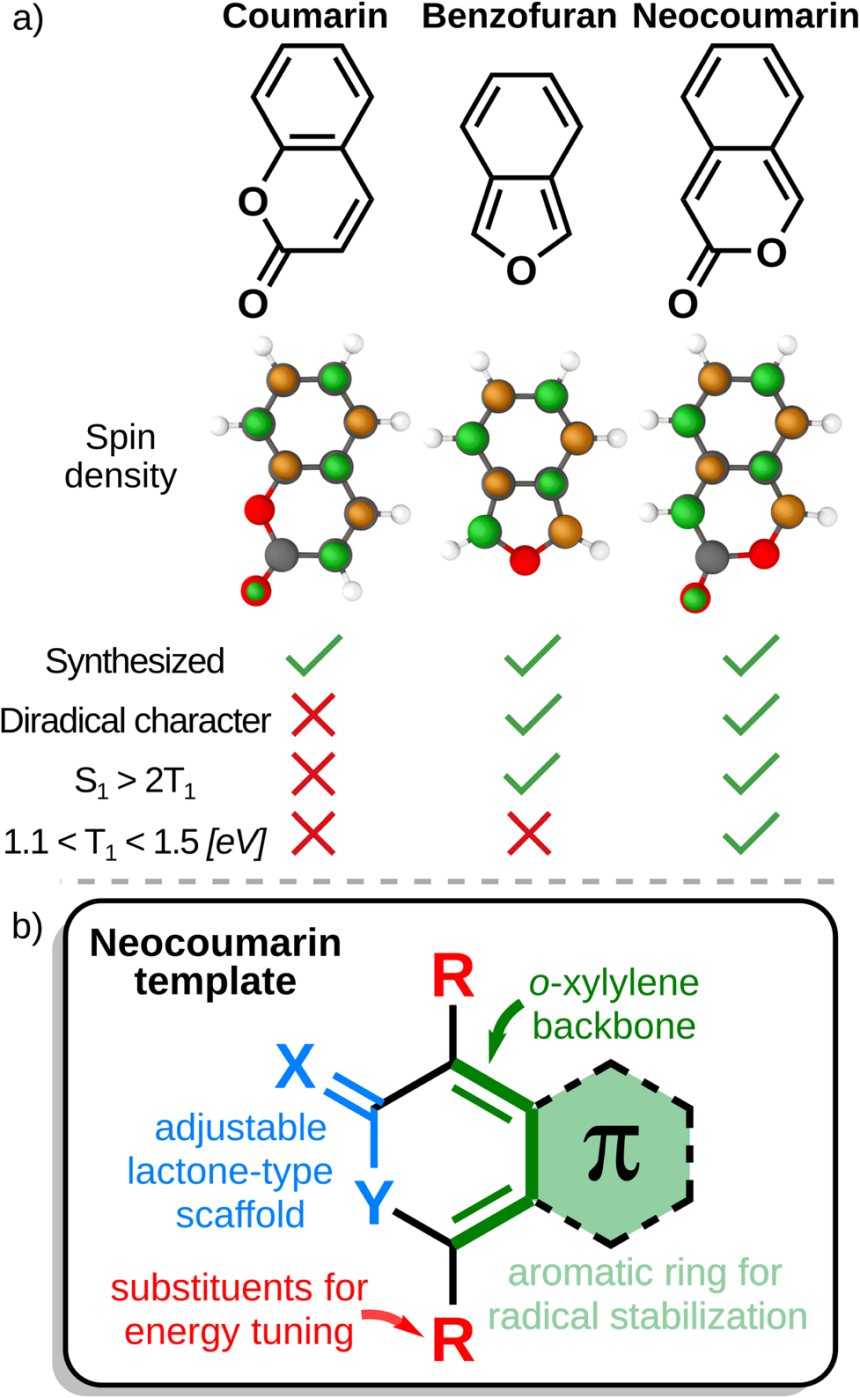
(a) Spin-density plots and comparison of coumarin, benzofuran, and neocoumarin. (b) Components of the neocoumarin scaffold. The *ortho* quinoid motif responsible for SF-relevant properties is highlighted in green.

We note the similarity between the neocoumarin core and the previously identified 2-benzofuran derivatives pioneered by Michl and coworkers (Fig. 9a).^17,106–109^ In both cases, the presence of *o*-xylylene motif characterized by two *ortho* quinoid endocyclic double bonds appears to be conducive to good SF energies. As expected from this analysis, removing this motif by altering aromaticity (as in 27 and 28) or by changing the position of the ester functional group in the 2-pyrone ring (Fig. 8), disturbs the energetics of the system.

In the case of 2-benzofuran, the presence of the *o*-xylylene motif (dark green in Fig. 9b), has been linked with diradicaloid character, an electronic property associated with SF propensity.^100,103,104,110,111^ To assess whether the same applies to the identified class of molecules, we evaluated the di- and tetraradical character (*y*_0_ and *y*_1_, respectively) of neocoumarin and selected derivatives *via* natural orbital analysis of their unrestricted Hartree–Fock (UHF) wavefunctions (see SI for additional analysis).^110,112,113^ The results are compiled in Fig. 9. We find that the diradical character in neocoumarin (*y*_0_ = 0.16) is slightly higher than in benzofuran (*y*_0_ = 0.09, hence the lower T_1_ energy of the former, *cf.*Fig. 8) while both retain very small tetraradical character (*y*_1_ = 0.01). By comparison, coumarin has little of both diradical and tetraradical character (*y*_0_ = 0.02, *y*_1_ = 0.00). These findings, along with the features of the spin density plots (Fig. 9) highlight the critical role of the *ortho* quinoid arrangement and the necessity of preserving aromaticity in maintaining singlet fission (SF)-relevant electronic properties, offering valuable insights for the design of next-generation SF molecules.

## 4 Conclusions

In this work, we demonstrated the relevance of a data-driven generative framework for the discovery of potential SF materials, combining structure generation and property prediction models. Building upon the FORMED database and leveraging curriculum-based reinforcement learning, this approach successfully rediscovered a broader range of SF chromophores than our previous fragment-based design methods, including polyenes, acenes, boron-dipyrromethane (BODIPY), benzofurans, fulvenoids, quinoidal structures, and mesoionic compounds. More significantly, the generative framework identified a molecular class, neocoumarins (2-benzopyran-3-one), which is uncharted for optoelectronic applications but exhibits favorable excited-state energetics for SF. While the coumarin and isocoumarin systems, well-represented in the FORMED database, exhibited poor SF properties, the generative model, guided by our RL optimization strategy, uncovered this third coumarin isomer, which is absent from the FORMED database but is found to possess promising SF energetics. This *ortho*-xylylene core capped by a cyclic ester group follows Michl's diradical design principles while addressing some of the limitations of the benzofuran core and offering novel opportunities for SF material development.

Our results thus highlight the potential of generative design not only to rediscover known SF candidates but also to explore uncharted regions of chemical space, enabling the identification of out-of-the-box chromophores with tailored properties. This offers a promising pathway for advancing the discovery of functional materials. Although we successfully identified a variety of target molecules, our current approach relies on manually guiding the generation process by excluding previously discovered scaffolds. This process could be streamlined by automatically detecting key scaffolds identified at the end of each trial and dynamically updating the list of excluded motifs for subsequent trials. Furthermore, direct comparisons with other approaches (*e.g.*, genetic optimization) across a broader range of practical chemical applications are still awaited.

## Author contributions

T. W. proposed the project. T. W. developed the generative design pipeline and performed all experiments. T. W., R. L., and J. T. B. analyzed the data and wrote the initial draft of the manuscript. C. C. guided the research direction and supervised the project, provided funding, edited and modified the content of the manuscript.

## Conflicts of interest

There are no conflicts to declare.

## Supplementary Material

SC-016-D5SC03184B-s001

## Data Availability

The codes for training the generative models and performing the reinforcement learning, which include all the scripts, the configuration files, the pre-RL and post-RL generative models, and the Chemprop excited-state property prediction model, are available at GitHub: https://github.com/lcmd-epfl/SF_generative. The FORMED database, used to train the models, is publicly available in the Materials Cloud Archive at https://doi.org/10.24435/materialscloud:nh-gb. The SI contains the Chemprop model performance metrics, the definitions of the scoring functions used for the reinforcement learning, the implementation details related to the training of the generative model and to the curriculum-based optimization, the complete results for all optimization trials, the additional reinforcement learning runs targeting unexplored scaffolds, the computed excited-state properties and the Chemprop prediction errors for the candidate molecules as well as further excited-state analyses of coumarin, isocoumarin, and neocoumarin derivatives. Supplementary information is available. See DOI: https://doi.org/10.1039/d5sc03184b.
