# Supplementary material for: Generative design of singlet fission materials leveraging a fragment-oriented database
Source: Chem Sci. 2025 Aug 25;16(38):17956–69. doi: 10.1039/d5sc03184b (PMC12418038; doi:10.1039/d5sc03184b)
Supplement: SC-016-D5SC03184B-s001 [file SC-016-D5SC03184B-s001.pdf]

Supplementary Information (SI) for Chemical Science.
This journal is © The Royal Society of Chemistry 2025

Electronic Supplementary Information for "Generative Design of Singlet Fission Materials Leveraging a Fragment-Oriented Database"

Thanapat Worakul,[†] Rubén Laplaza,[†,‡] J. Terence Blaskovits,[†,¶] and Clémence Corminboeuf*,[†,‡]

†*Laboratory for Computational Molecular Design, Institute of Chemical Sciences and Engineering, Ecole Polytechnique Fédérale de Lausanne (EPFL), 1015 Lausanne, Switzerland*

‡*National Center for Competence in Research-Catalysis (NCCR-Catalysis), École Polytechnique Fédérale de Lausanne (EPFL), 1015 Lausanne, Switzerland*

¶*Max-Planck Institute for Polymer Research, Ackermannweg 10, 55128 Mainz, Germany*

E-mail: clemence.corminboeuf@epfl.ch

Table of Contents

S1: Chemprop Model Performance **3**

S2: Score Functions **4**

S3: Generative Model Training and Reinforcement Learning **9**

S4: All Optimization Trial Results **10**

S5: Additional Optimization Trials **17**

S6: Adiabatic Excited-State Energies and Chemprop Prediction Errors of Candidate Molecules **19**

S7: Additional Results for Coumarin, Isocoumarin, and Neocoumarin Structures **22**

S1: Chemprop Model Performance

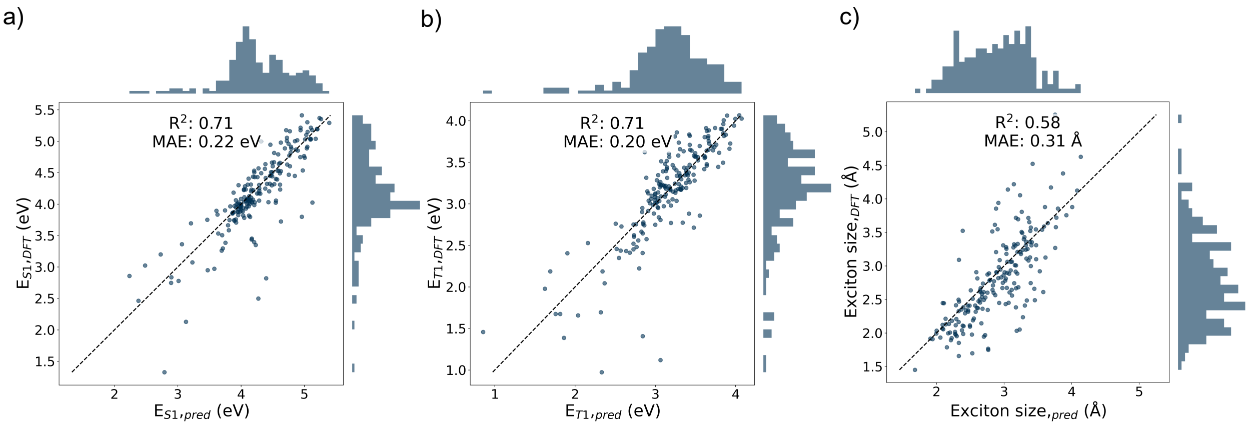

Figure S1: Correlation plots of excited-state properties comparing the predicted values from the Chemprop model with the true values for molecules in the external test set, taken from the previous work:[1] a) $E_{S_1,\mathrm{ve}}$, b) $E_{T_1,\mathrm{ve}}$, and c) $d^{S_1}_{e^- - h^+}$.

S2: Score Functions

The energy score function, as formulated in previous work,[1] is utilized to optimize for the SF energetic criteria. This score is defined as the minimum signed distance to the boundary of the blue triangular region, defined by the points (1.5 eV, 3.8 eV), (1.9 eV, 3.8 eV), and (1.5 eV, 3.0 eV), where $E_{T_{1,ve}}$ and $E_{S_{1,ve}}$ values satisfy the vertical SF energetic requirements (Figure S3).

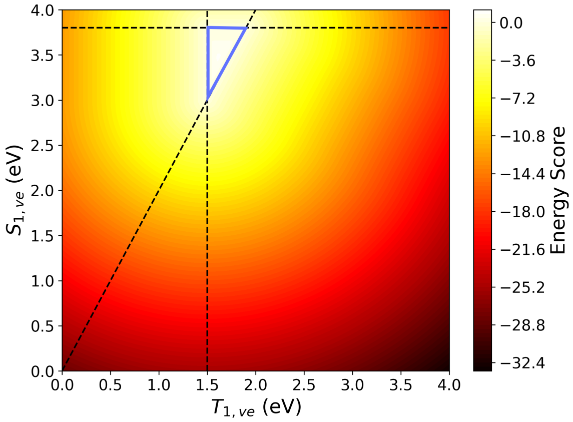

Figure S2: Energy score as a function of S_1 and T_1 for evaluating vertical (Franck-Condon) singlet fission (SF) properties. The target region, where molecules satisfy SF energetic requirements, is enclosed within the blue triangular area, corresponding to where the energy score value is positive. The dashed lines indicate the boundaries defined by the conditions: $S_{1,ve}$ - $2T_{1,ve} > 0$ eV, $S_{1,ve} = 3.8$ eV, and $T_{1,ve} = 1.5$ eV. The vertical dotted line represents the minimum $T_{1,ve}$ of 1.5 eV, a threshold for utilizing SF materials as multiexciton-generating components in Si solar cells, extrapolated from an adiabatic-vertical energy scaling relationship.[2,3] The horizontal dashed line at $S_{1,ve} = 3.8$ eV accounts for the solar energy spectrum distribution.

In an event that (S_1,T_1) lies inside the triangle, the energy score is

$$E(T_1, S_1) = \min \begin{Bmatrix} \alpha \cdot d_1(T_1, S_1), \\ \alpha \cdot d_2(T_1, S_1), \\ \alpha \cdot \beta \cdot d_3(T_1, S_1) \end{Bmatrix}$$

with $\alpha = \frac{1}{0.11871871871871865} \approx 8.42$, $\beta = \frac{1}{3}$ and distances defined as:

$$d_1 = \text{distance to vertical line } T_1 = T_{\text{cut}} = |T_1 - T_{\text{cut}}|$$
$$d_2 = \text{distance to line } S_1 = 2T_1 = \frac{|2T_1 - S_1|}{\sqrt{5}}$$
$$d_3 = \text{distance to horizontal line } S_1 = S_{\text{cut}} = |S_1 - S_{\text{cut}}|$$

If the energy pair lies outside the triangle, the score becomes a negative value reflecting the Euclidean distance to the nearest point on the triangle defined by the vertices:

$$A = (T_{\text{cut}}, 2T_{\text{cut}}, 0) = (1.5, 3.0, 0)$$
$$B = (T_{\text{cut}}, S_{\text{cut}}, 0) = (1.5, 3.8, 0)$$
$$C = \left(\frac{S_{\text{cut}}}{2}, S_{\text{cut}}, 0\right) = (1.9, 3.8, 0)$$

Let $P = (T_1, S_1, 0)$, the energy score is defined as:

$$E(S_1, T_1) = -\alpha \cdot \text{distance from } P \text{ to } \triangle ABC$$

To ensure structural diversity in the generated molecules, we implement a diversity filter that penalizes redundant topological scaffolds. Generated scaffolds are stored in a bucket of size 30, and a penalty factor of 0.4 is applied to reduce the score of molecules containing previously generated scaffolds.

To further refine the generated molecules, we enforce constraints to avoid specific substructures represented as SMARTS strings (custom alerts), which are listed below:

1. `[*;r8]`
2. `[*;r9]`
3. `[*;r10]`
4. `[*;r11]`
5. `[*;r12]`
6. `[*;r13]`

7. [*;r14]
8. [*;r15]
9. [*;r16]
10. [*;r17]
11. [C;!R]=[C;!R]~[C;!R]=[C;!R]~[C;!R]=[C;!R]
12. [C;!R]=[C;!R]~[C;!R]=[C;!R]~[C;!R]
13. [*;r]-[C;!R]=[C;!R][*;r]
14. [*;r]-[C;!R]=[C;!R] [C;!R]=[C;!R][*;r]
15. [6;R]=[6;!R][6;!R]=[6]

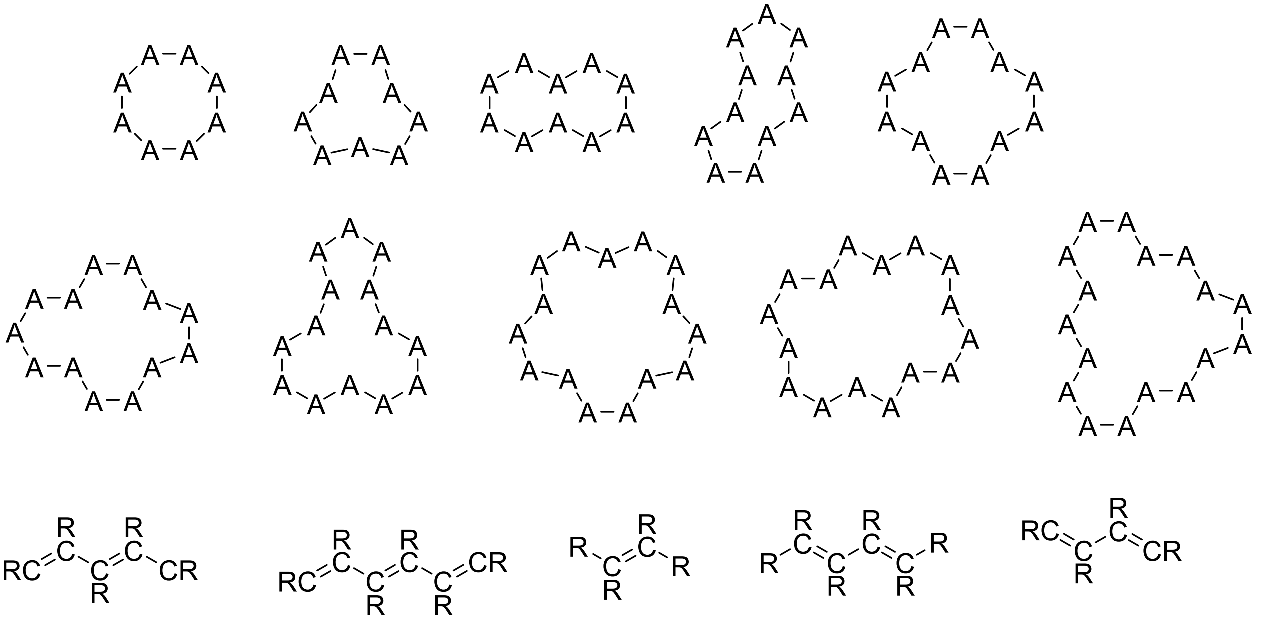

The score value for molecules containing any of these substructures is set to 0, ensuring that the generated molecules exclude these motifs toward the end of the optimization process. Furthermore, to enforce charge neutrality, a penalty is applied to charged molecules by multiplying their score by 0 (Enforce neutrality). We also incorporate the synthetic accessibility score (SAScore)[4] to bias the agent toward generating molecules that are more likely to be synthetically feasible.

The individual components of the score function are aggregated using a weighted geometric mean:

$$\mathbf{S}(T) = \left(\prod_{i=1}^{n} \mathbf{S}i(T)^{w_i} \right)^{\frac{1}{\sum i=1^n w_i}} \tag{1}$$

The weights assigned to each component at different stages of curriculum learning (CL) are as follows:

Table S1: Scoring components and weights in Eq. 1 used in the first and second stages of RL optimization.

	**Component**	**Weight**
First stage	Custom alerts	1
	SAScore	0.5
	Enforce neutrality	1
Second stage	Custom alerts	1
	Energy score	1.3
	Exciton size	0.5
	$T_2 - T_1$ energy gap	0.4

To ensure all score components fall within the range [0,1], we apply sigmoid and reverse sigmoid transformations to components whose raw values lie outside this interval, following the implementation in REINVENT4.[5] The transformations are defined as follows:

$$S_{\text{reverse}}(x) = 1 - \sigma\left(\frac{10k}{\text{high} - \text{low}} \left(x - \frac{\text{high} + \text{low}}{2} \right) \right)$$

$$\sigma(h) = \begin{cases} \dfrac{1}{1 + e^{-h}}, & h \geq 0, \\ \dfrac{1}{1 + e^{h}}, & h < 0. \end{cases}$$

$$S(x) = \sigma\left(\frac{10k}{\text{high} - \text{low}} \left(x - \frac{\text{high} + \text{low}}{2} \right) \right)$$

where the sigmoid function $\sigma(h)$ is evaluated using a numerically stable formulation:

$$\sigma(h) = \begin{cases} \dfrac{1}{1+e^{-h}}, & h \geq 0, \\ \dfrac{1}{1+e^{h}}, & h < 0. \end{cases}$$

The hyperparameters used for each score component are summarized in Table S2.

Table S2: Score component transformations and associated hyperparameters.

Score component	Transformation	k	high	low
Custom alert	None	–	–	–
SAscore	Reverse sigmoid	0.4	10	1
SF energy score	Sigmoid	0.4	5	-10
Exciton size	Sigmoid	0.4	10	0
T2–T1 gap	Sigmoid	0.4	10	0

S3: Generative Model Training and Reinforcement Learning

The generative model was trained using REINVENT v3.2[6] on all canonical SMILES in the FORMED[7] database. The model is configured to accommodate a maximum sequence length of 356 tokens. We employ a Long Short-Term Memory (LSTM)[8] network with 3 layers, each having a layer size of 512 and an embedding layer size of 256. Each layer is normalized, and no dropout is applied. The training is carried out for 30 epochs with an initial learning rate of 0.0005 and a batch size of 128 molecules.

The generative model trained with REINVENT v3.2[6] is then converted for compatibility with REINVENT v4[9] using the conversion script provided in the REINVENT4 GitHub repository.[5] Reinforcement learning (RL) is carried out by initializing both the prior and agent networks with the trained generative model described above. During each RL stage, we employ a batch size of 128 and a learning rate of 0.001. The first stage of the curriculum is run for 20 iterations, while the second stage is extended to 400–700 iterations.

The learning objective of RL in each stage is formulated as the Difference between Augmented and Posterior (DAP) loss,[10] given by:

$$\begin{aligned} \mathcal{L}(T) &= \left(\log \mathbf{P}_{\text{aug}}(T) - \log \mathbf{P}_{\text{agent}}(T)\right)^2 \\ \log \mathbf{P}_{\text{aug}}(T) &= \log \mathbf{P}_{\text{prior}}(T) + \sigma \mathbf{S}(T) \end{aligned} \tag{2}$$

where $\mathbf{S}(T)$ is the reward value associated with the SMILES string T, and σ is a scaling factor set to 256. $\mathbf{P}_{\text{agent}}(T)$ is the likelihood of SMILES sequence T in the agent network, and $\mathbf{P}_{\text{prior}}(T)$ is the likelihood of SMILES sequence T in the prior network.

S4: All Optimization Trial Results

Table S3: Summary of generative model performance. For each model, 1,280 molecules were created with the generative model in three independent trials. The mean and standard deviation of key excited-state properties across these trials are reported. These include the energy score (to be maximized), which evaluates SF criteria as shown in Figure S2; the exciton size (i.e., the root-mean-square electron-hole separation, to be maximized), which promotes delocalized singlet exciton formation; and the energy gap between the vertical second and first triplet excited states, $E_{T_2,ve} - E_{T_1,ve}$ and $E_{T_2,ve} - 2E_{T_1,ve}$ (to be maximized), to reduce the likelihood of the T_1 to T_2 upconversion competing processes. The number of unique scaffolds is defined as Murcko scaffolds with a Tanimoto similarity below 0.7 relative to other scaffolds in the set.

Generative Model	Energy Score	Exciton Size (Å)	T_2-T_1 (eV)	T_2-$2T_1$ (eV)	Unique Scaffolds	Similarity Score
Pre-RL	-16.47 ± 0.35	2.78 ± 0.12	0.54 ± 0.35	-3.11 ± 0.13	367 ± 80	0.10 ± 0.03
Post-RL 1 (no custom alert)	-0.56 ± 0.08	5.22 ± 0.01	1.20 ± 0.08	-0.59 ± 0.02	2 ± 1	0.35 ± 0.01
Post-RL 2 (with custom alert)	-1.37 ± 0.05	4.47 ± 0.01	1.48 ± 0.12	-0.03 ± 0.03	2 ± 1	0.35 ± 0.02
Post-RL 3 (with custom alert)	-0.72 ± 0.04	3.65 ± 0.01	1.38 ± 0.08	-0.24 ± 0.04	2 ± 1	0.39 ± 0.01
Post-RL 4 (with custom alert)	-3.11 ± 0.11	3.98 ± 0.01	1.58 ± 0.13	-0.24 ± 0.09	15 ± 7	0.32 ± 0.02
Post-RL 5 (with custom alert)	-1.87 ± 0.10	4.32 ± 0.02	1.03 ± 0.12	-0.63 ± 0.02	6 ± 2	0.37 ± 0.02
Post-RL 6 (with custom alert)	-3.02 ± 0.07	3.94 ± 0.05	1.24 ± 0.27	-0.77 ± 0.04	65 ± 30	0.23 ± 0.01
Post-RL 7 (with custom alert)	-2.02 ± 0.03	3.40 ± 0.01	1.42 ± 0.27	-0.86 ± 0.05	2 ± 1	0.34 ± 0.02
Post-RL 8 (with custom alert)	-1.97 ± 0.08	4.37 ± 0.00	1.17 ± 0.19	-0.69 ± 0.01	5 ± 2	0.27 ± 0.01
Post-RL 9 (with custom alert)	-1.67 ± 0.05	3.80 ± 0.01	1.12 ± 0.21	-0.36 ± 0.14	3 ± 1	0.39 ± 0.01
Post-RL 10 (with custom alert)	-3.86 ± 0.01	4.10 ± 0.01	1.02 ± 0.13	-0.79 ± 0.02	1 ± 0	0.33 ± 0.01

Table S4: Key generative model quality metrics: percentage of chemically valid molecules (validity), proportion of unique structures (uniqueness), fraction of molecules not seen in the training set (novelty), and hit-rate of molecules meeting SF criteria. For each model, 1,280 molecules were created with the generative model in three independent trials. The mean and standard deviation of key excited-state properties across these trials are reported.

Generative Model	Validity (%)	Uniqueness (%)	Novelty (%)	Hit-Rate (%)
Pre-RL	93.3 ± 0.6	100.0 ± 0.0	98.0 ± 0.8	2.15 ± 0.4
Post-RL 1	87.3 ± 1.9	100.0 ± 0.0	97.0 ± 0.1	95.4 ± 1.0
Post-RL 2	91.5 ± 1.2	100.0 ± 0.0	100.0 ± 0.0	98.1 ± 0.1
Post-RL 3	95.5 ± 0.8	100.0 ± 0.0	99.3 ± 0.5	99.5 ± 0.4
Post-RL 4	65.8 ± 0.9	100.0 ± 0.0	99.7 ± 0.5	77.4 ± 1.0
Post-RL 5	83.3 ± 0.1	100.0 ± 0.0	99.7 ± 0.5	96.3 ± 0.2
Post-RL 6	79.6 ± 1.7	100.0 ± 0.0	99.7 ± 0.5	82.4 ± 2.0
Post-RL 7	68.5 ± 1.7	100.0 ± 0.0	98.3 ± 0.5	73.6 ± 0.1
Post-RL 8	86.1 ± 1.2	100.0 ± 0.0	100.0 ± 0.0	88.9 ± 0.5
Post-RL 9	85.4 ± 0.6	100.0 ± 0.0	100.0 ± 0.0	94.1 ± 0.5
Post-RL 10	80.1 ± 1.5	100.0 ± 0.0	100.0 ± 0.0	79.6 ± 1.0

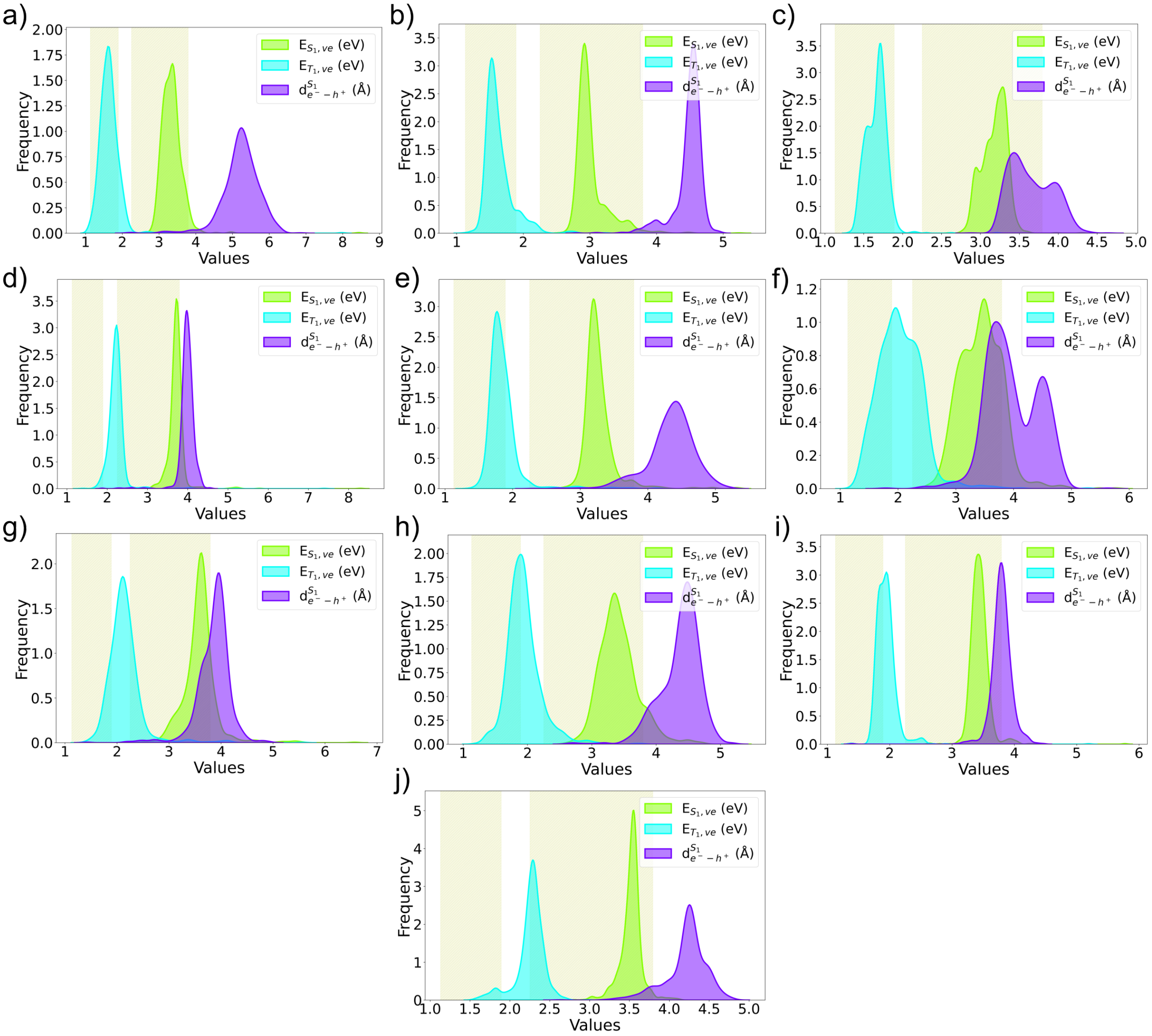

Figure S3: Kernel density histogram plots of $E_{S_1,ve}$, $E_{T_1,ve}$, and $d^{S_1}_{e^- - h^+}$ predicted with the Chemprop model for 1280 molecules generated from the post-RL generative models: (a) 1, (b) 2, (c) 3, (d) 4, (e) 5, (f) 6, (g) 7, (h) 8, (i) 9, and (j) 10. The yellow region designates where $S_1 \rightarrow 2T_1$ conversion is thermodynamically feasible and $E_{T_1,ve}$ is aligned for potential integration into solar cell applications.

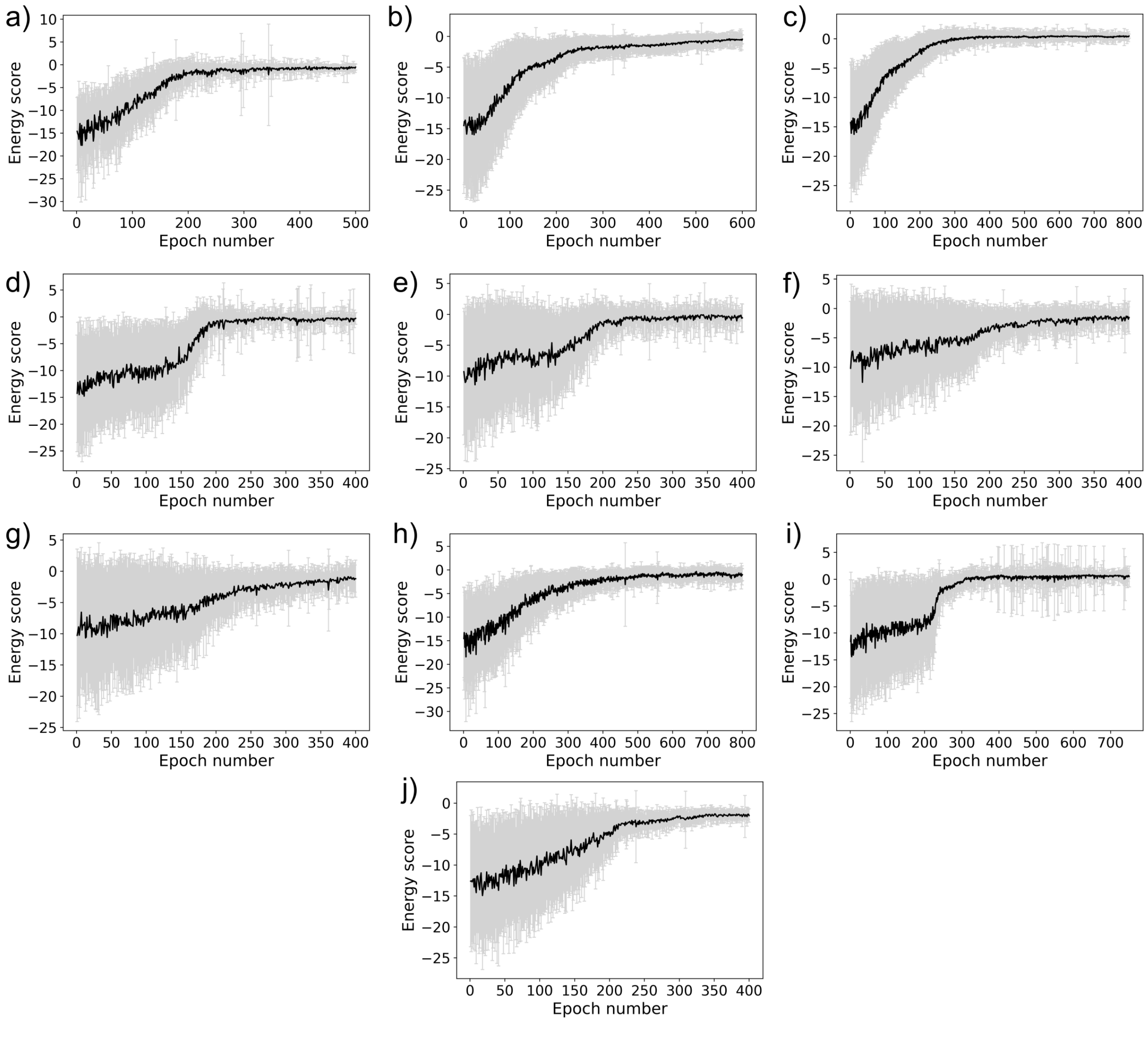

Figure S4: RL optimization curves during stage 2 of the curriculum for the energy score in the trial number: (a) 1, (b) 2, (c) 3, (d) 4, (e) 5, (f) 6, (g) 7, (h) 8, (i) 9, and (j) 10.

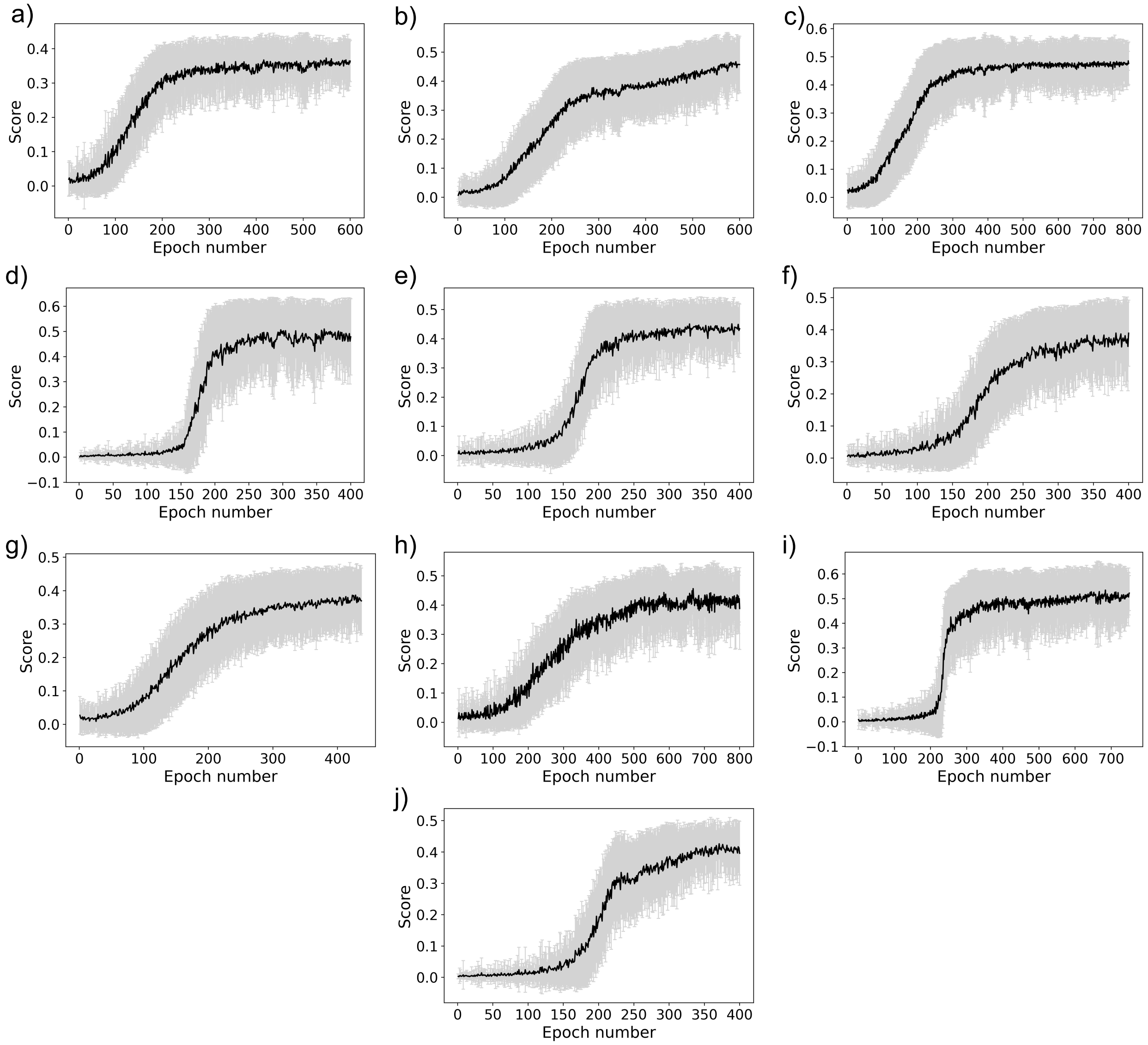

Figure S5: RL optimization curves during stage 2 of the curriculum for the overall score in the trial number: (a) 1, (b) 2, (c) 3, (d) 4, (e) 5, (f) 6, (g) 7, (h) 8, (i) 9, and (j) 10.

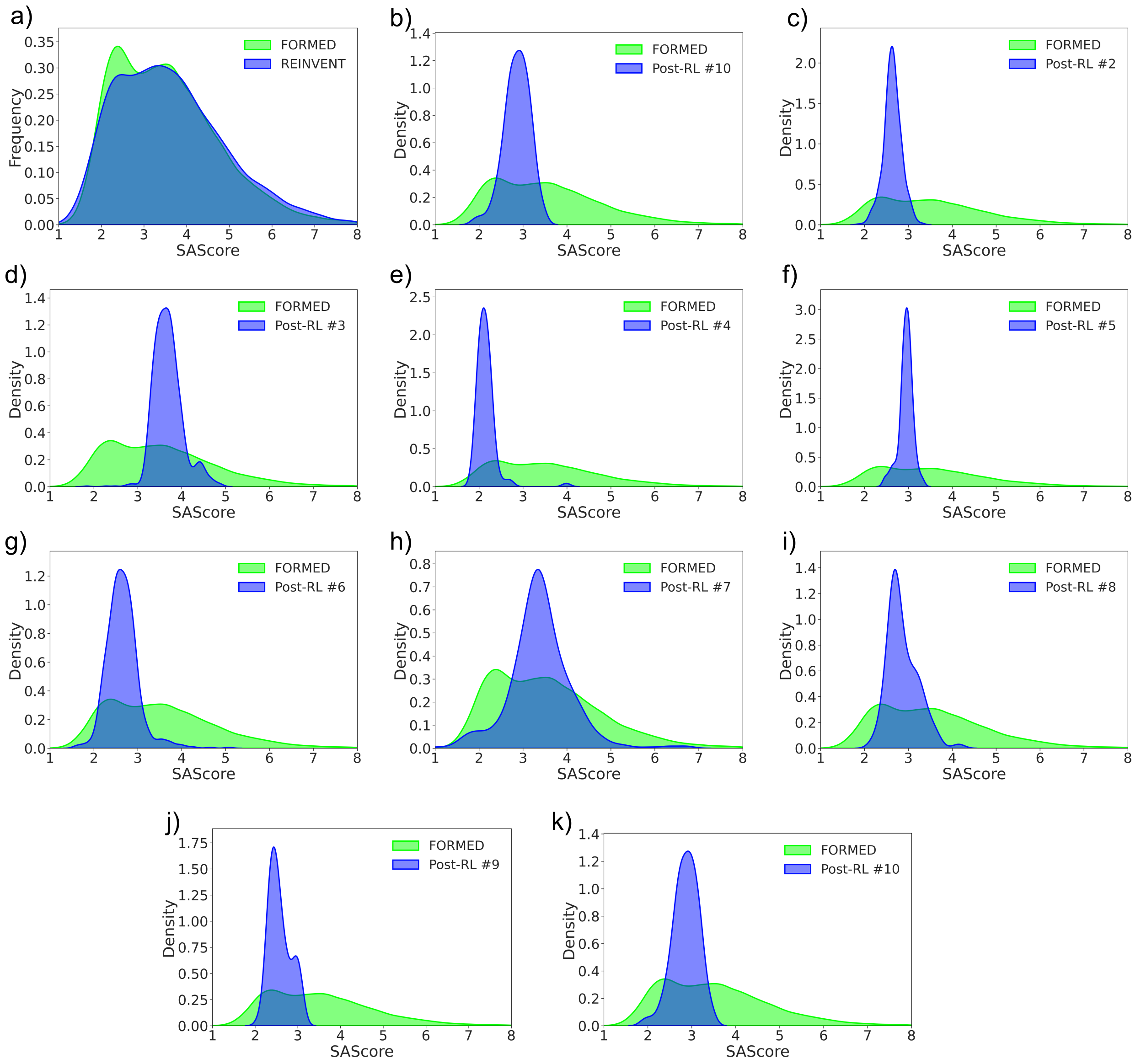

Figure S6: Kernel density histogram plots of SAScore for molecules in FORMED and the generated molecules with the generative models: (a) pre-RL model, (b) post-RL model 1, (c) post-RL model 2, (d) post-RL model 3, (e) post-RL model 4, (f) post-RL model 5, (g) post-RL model 6, (h) post-RL model 7, (i) post-RL model 8, (j) post-RL model 9 and (k) post-RL model 10.

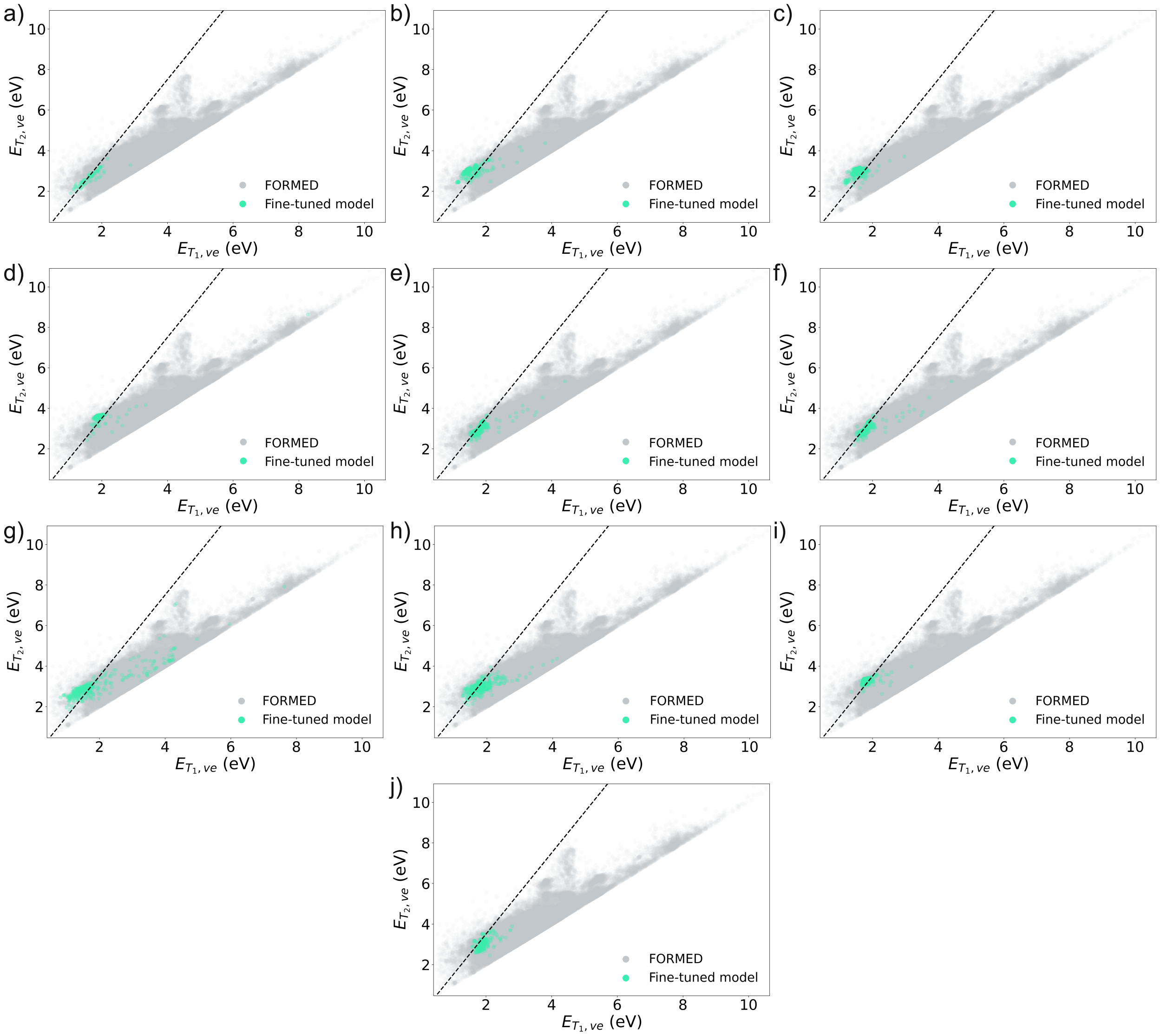

Figure S7: Property map between $E_{T_1,ve}$ and $E_{T_2,ve}$ of FORMED database (gray) overlaid on top of 1280 generated structures from (a) post-RL model 1, (b) post-RL model 2, (c) post-RL model 3, (d) post-RL model 4, (e) post-RL model 5, (f) post-RL model 6, (g) post-RL model 7, (h) post-RL model 8, (i) post-RL model 9 and (j) post-RL model 10. The dashed line indicates where $E_{T_2,ve}$ - $2E_{T_1,ve}$ = 0.5 eV

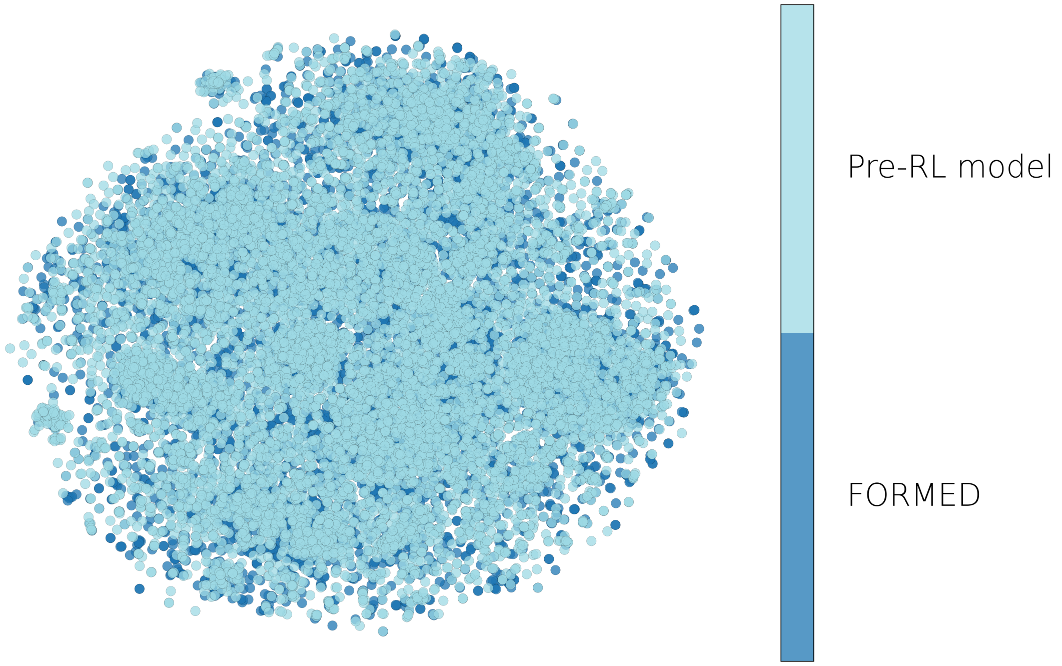

Figure S8: t-SNE plot generated from the Morgan fingerprint representation of the FORMED dataset and generated structures from the pre-RL generative models.

S5: Additional Optimization Trials

Following the appearance of the neocoumarin scaffold in the 10th trial, we conducted additional RL trials, adding the neocoumarin scaffold to the list of unwanted substructures. Using the same score functions and RL optimization configuration, these subsequent trials yielded scaffolds not identified in any of the previous run. This suggests that a portion of the SF-relevant chemical space remains unexplored after our ten RL optimization runs. We leave the automation of this iterative process of chemical space exploration to dynamically identify and exclude the already discovered motifs as future work, with the goal of uncovering a more comprehensive set of potential SF scaffolds.

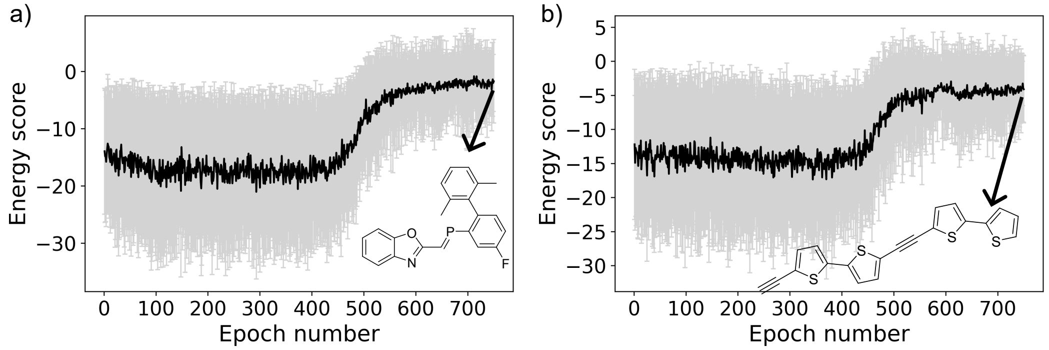

Figure S9: RL optimization curves during stage 2 of the curriculum for the energy score in the two additional trial numbers, along with the representative discovered SF molecules.

To identify neocoumarin derivatives through RL optimization, we incorporated a similarity score to the neocoumarin scaffold into the scoring function. This guided the generative model in creating neocoumarin-like molecules that exhibit favorable SF properties. The resulting structures feature a 2-pyrone core fused with various aromatic systems, some of which had also been identified in our manual systematic exploration of neocoumarin derivatives (e.g., fused rings linking a lactone and a heterocycle). Notably, the local chemical space exploration also yielded fused tricyclic ring systems. However, these candidates have T1 energy levels that are too low for the semiconductor band gap.

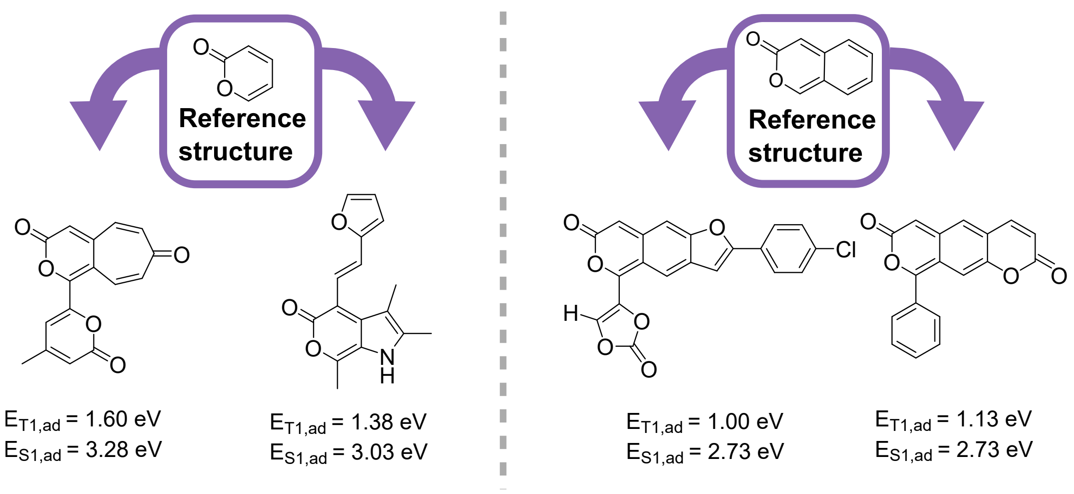

Figure S10: Identified neocoumarin derivatives from the RL trials where the similarity score to the reference structure is incorporated into the score function.

S6: Adiabatic Excited-State Energies and Chemprop Prediction Errors of Candidate Molecules

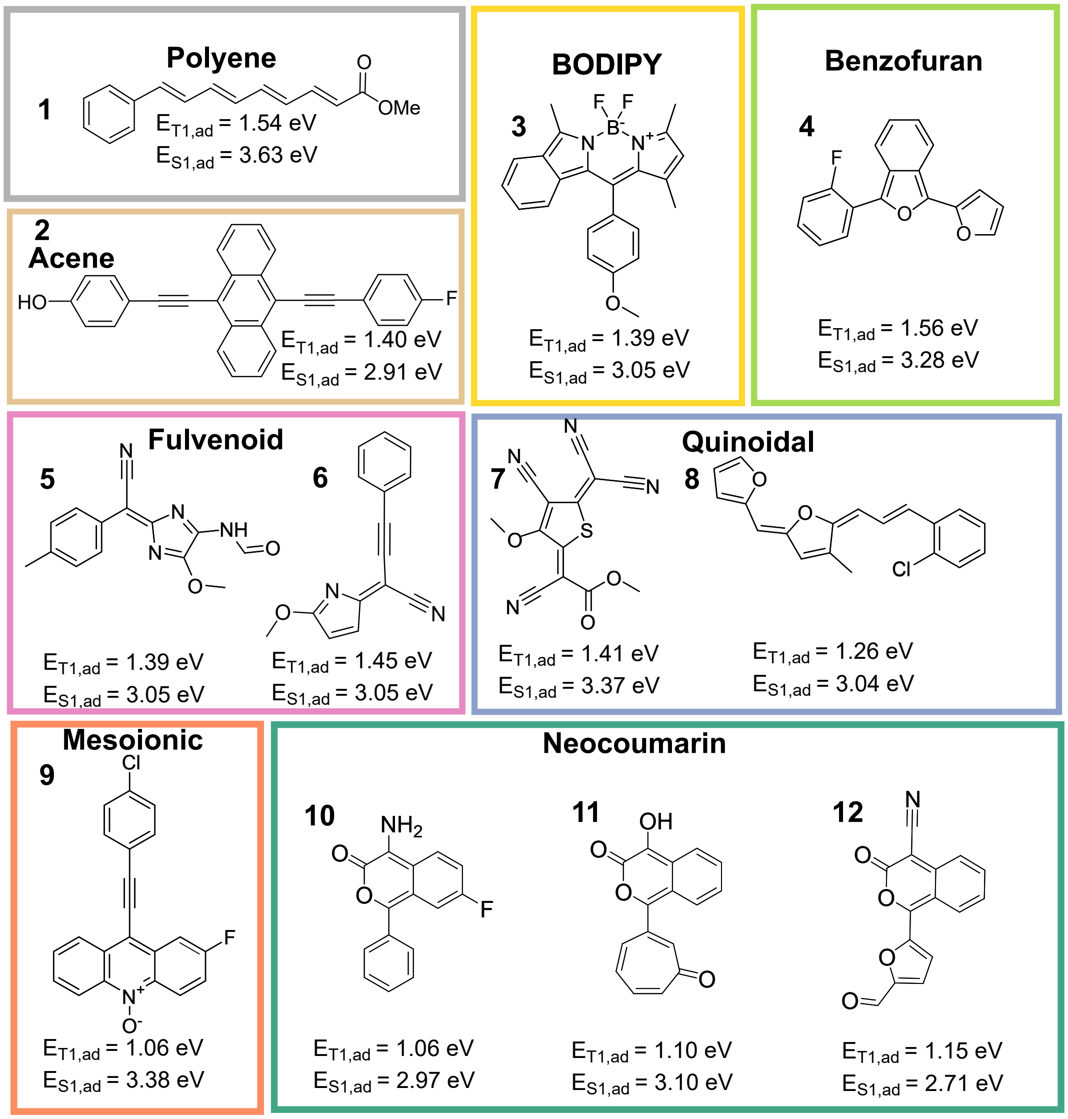

Figure S11: Candidate molecules generated by the post-RL generative models from different optimization trial runs. $E_{T_1,ad}$ and $E_{S_1,ad}$ are computed using adiabatic TD-DFT.

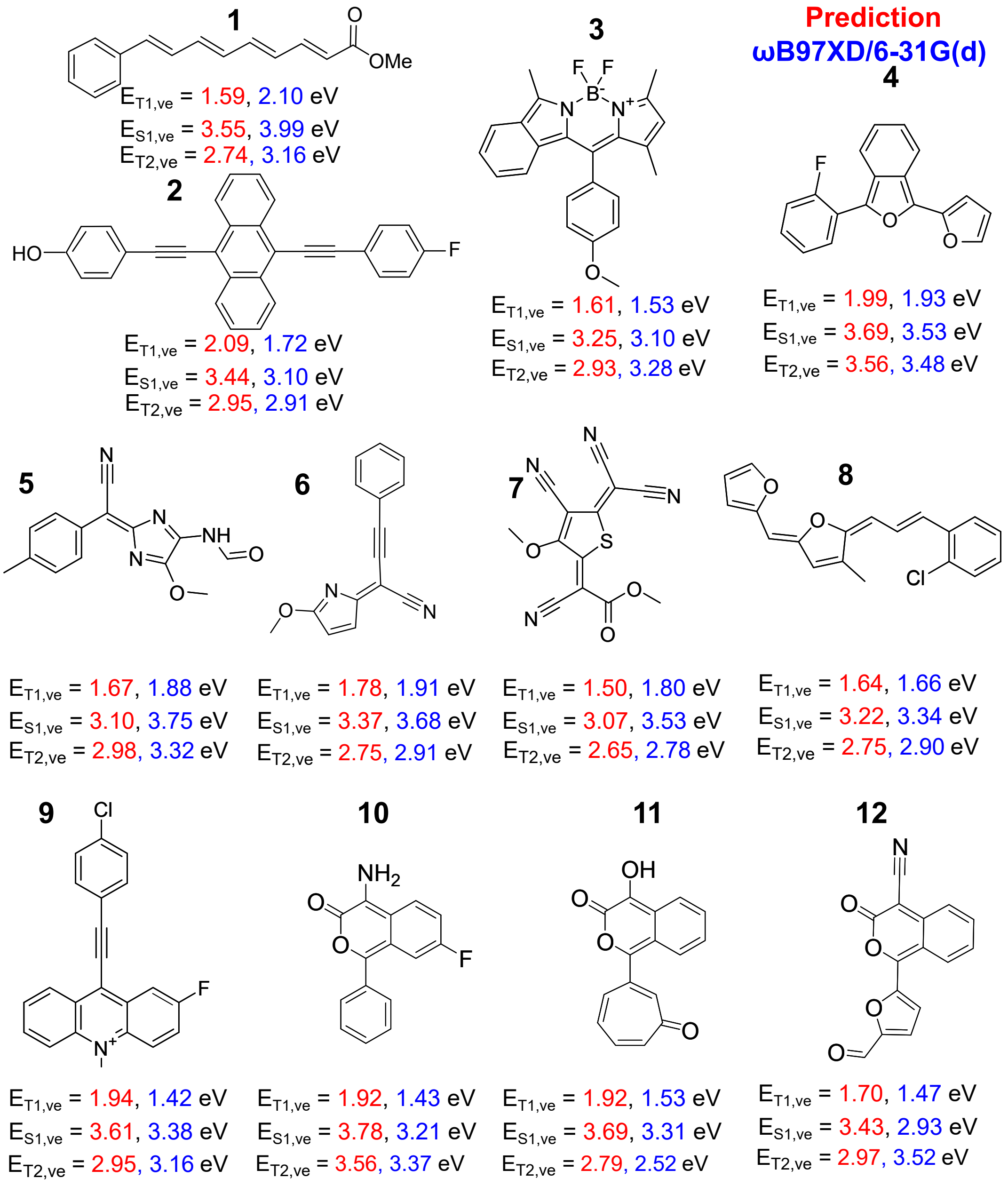

Figure S12: Predicted (red) and DFT-computed (blue) vertical excited-state energies for the candidate molecules generated by the post-RL generative models from different optimization trial runs.

Table S5: Root-mean-square deviation (RMSD) and mean absolute error (MAE) in eV of the trained Chemprop model, evaluated on 100 candidate molecules, computed at ωB97XD/6-31G(d), from 10 reinforcement learning optimization trials.

	**RMSD (eV)**	**MAE (eV)**
$E_{S_1,ve}$	0.45	0.31
$E_{T_1,ve}$	0.41	0.28
$E_{T_2,ve}$	0.23	0.30

S7: Additional Results for Coumarin, Isocoumarin, and Neocoumarin Structures

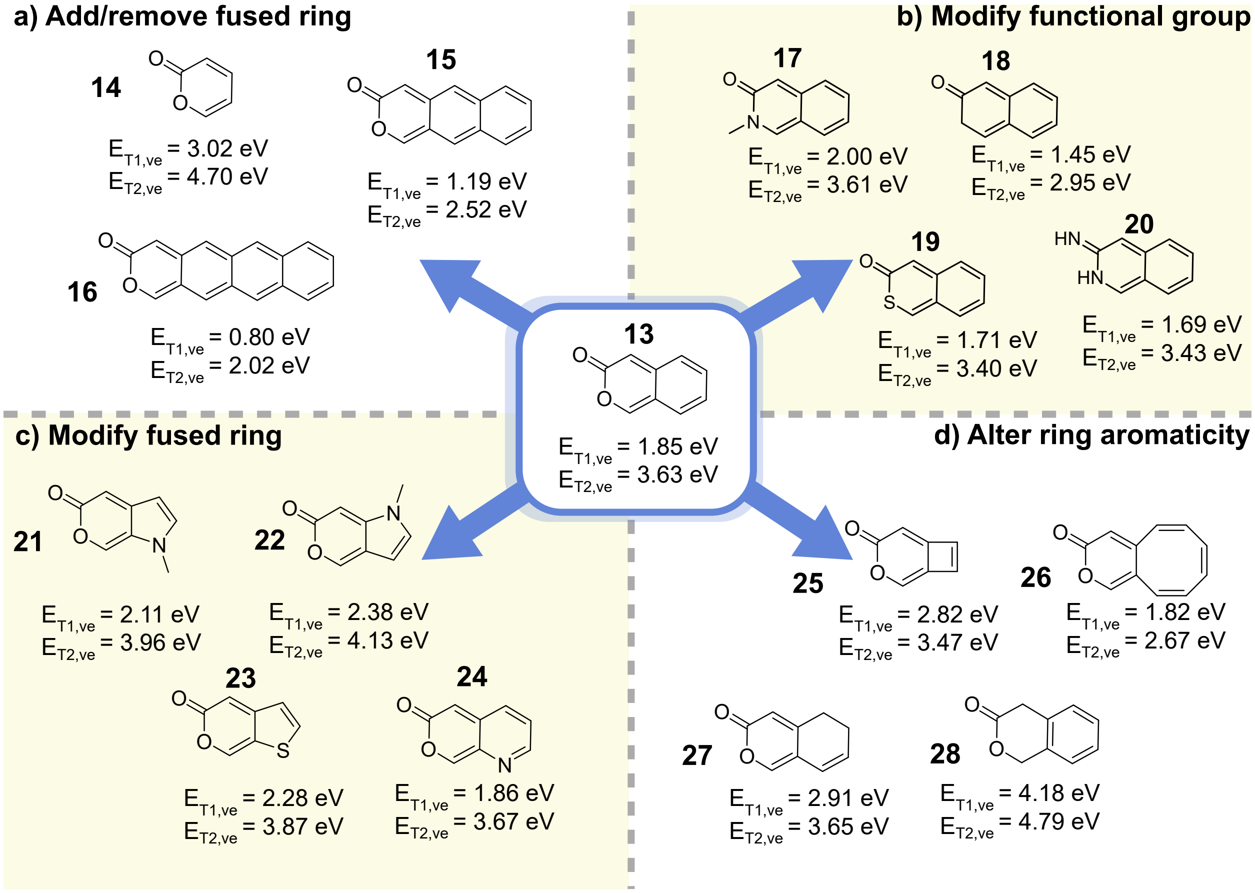

Figure S13: Derivative structures of neocoumarin and their $E_{T_1,ve}$ and $E_{T_2,ve}$ from a) adding or removing fused rings, b) modifying the capping functional group, c) switching the benzene ring to other aromatic fused rings, and d) altering the aromaticity of the conjugated system. Structural changes that preserve excited-state energies that satisfy SF criteria are indicated by the yellow background

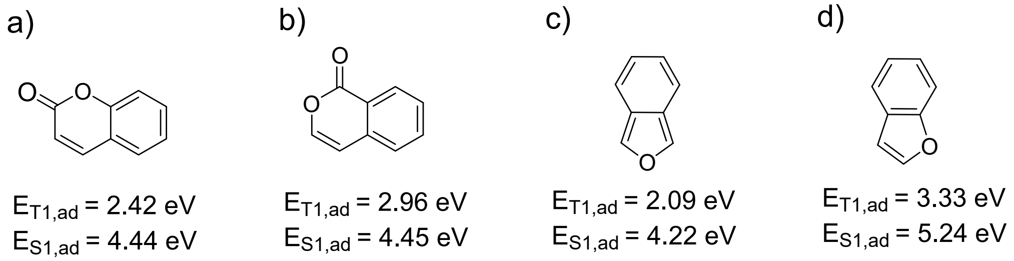

Figure S14: The adiabatic S_1 and T_1 excited state energies of a) coumarin, b) isocoumarin, c) 2-benzofurane, and d) 1-benzofurane

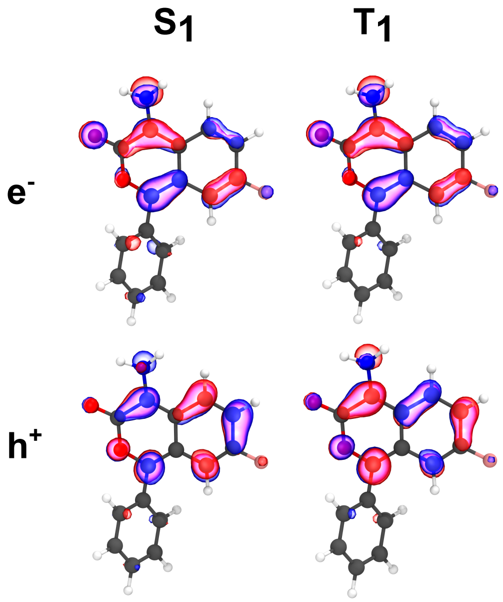

Figure S15: The natural transition orbitals (NTOs) of **10**, computed with Multiwfn software package[11]

References

(1) Schaufelberger, L.; Blaskovits, J. T.; Laplaza, R.; Jorner, K.; Corminboeuf, C. Inverse Design of Singlet-Fission Materials with Uncertainty-Controlled Genetic Optimization. *Angew. Chem. Int. Ed.* **2025**, *64*, e202415056.

(2) Blaskovits, J. T.; Fumanal, M.; Vela, S.; Cho, Y.; Corminboeuf, C. Heteroatom oxidation controls singlet–triplet energy splitting in singlet fission building blocks. *Chem. Commun.* **2022**, *58*, 1338–1341.

(3) Blaskovits, J. T.; Fumanal, M.; Vela, S.; Corminboeuf, C. Designing Singlet Fission Candidates from Donor–Acceptor Copolymers. *Chem. Mater.* **2020**, *32*, 6515–6524.

(4) Chen, S.; Jung, Y. Estimating the synthetic accessibility of molecules with building block and reaction-aware SAScore. *J. Cheminform.* **2024**, *16*, 83.

(5) REINVENT4. `https://github.com/MolecularAI/REINVENT4`.

(6) REINVENT 3.2. `https://github.com/MolecularAI/Reinvent`.

(7) Blaskovits, J. T.; Laplaza, R.; Vela, S.; Corminboeuf, C. Data-Driven Discovery of Organic Electronic Materials Enabled by Hybrid Top-Down/Bottom-Up Design. *Adv. Mater.* **2024**, *36*, 2305602.

(8) Hochreiter, S.; Schmidhuber, J. Long Short-Term Memory. *Neural Comput.* **1997**, *9*, 1735–1780.

(9) Loeffler, H. H.; He, J.; Tibo, A.; Janet, J. P.; Voronov, A.; Mervin, L. H.; Engkvist, O. Reinvent 4: Modern AI–driven generative molecule design. *J. Cheminform.* **2024**, *16*, 20.

(10) Fialková, V.; Zhao, J.; Papadopoulos, K.; Engkvist, O.; Bjerrum, E. J.; Kogej, T.; Patronov, A. LibINVENT: reaction-based generative scaffold decoration for in silico library design. *J. Chem. Inf. Model.* **2021**, *62*, 2046–2063.

(11) Lu, T.; Chen, F. Multiwfn: A multifunctional wavefunction analyzer. *J. Comput. Chem.* **2012**, *33*, 580–592.
